# Benzylic deuteration of alkylnitroaromatics via amine‐base catalysed exchange with deuterium oxide

**DOI:** 10.1002/jlcr.4008

**Published:** 2022-12-16

**Authors:** Stephen Maddocks, Nurul F. Samuri, Katerina Ridge, Ian D. Cunningham, William J. S. Lockley

**Affiliations:** ^1^ Department of Chemistry, Faculty of Engineering and Physical Sciences University of Surrey Guildford UK

**Keywords:** alkylnitroaromatics, amine‐base‐catalysis, deuteration, DNT, HIE, isotope exchange, nitrotoluenes

## Abstract

This paper describes the deuterium‐labelling of alkylnitroaromatics by base‐catalysed exchange with deuterium oxide. As the alkyl protons alpha to the aromatic ring are the most acidic sites in the molecule, regioselective hydrogen isotope exchange at this benzylic location leads to a regiospecifically deuterated product. The exchange labelling takes place in good yields and with high atom% abundance in the presence of an appropriate nitrogen base. Alkylated 2,4‐dinitrobenzenes deuterate at room temperature under catalysis by triethylamine, whilst alkylated 2‐nitro‐ or 4‐nitrobenzenes and related mono‐nitroaromatics require higher temperatures and catalysis by 1,5‐diazobicyclo[4.3.0]non‐5‐ene (DBN). The labelling reactions require an inert gas atmosphere, but otherwise are simple and high yielding with no obvious byproducts. Those compounds in which the benzylic protons are in an *ortho*‐orientation with respect to the nitro group label somewhat more slowly than the analogues where there is a *para* relationship. In addition, higher alkyl homologues undergo benzylic deuteration at slower rates than methyl.

## Graphical abstract

The alkyl groups of alkylnitroaromatics can be deuterated in the benzylic positions by isotopic exchange with deuterium oxide under catalysis by amine bases. Alkyldinitroaromatics label well at room temperature with triethylamine as catalyst, whereas mononitro sytems require a stronger base (DBN) and higher temperatures.

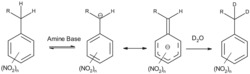

## 1 INTRODUCTION

Nitrated alkylaromatics are an important class of compounds and are utilised as intermediates in many high‐volume industrial processes. Typical markets for nitrotoluene feedstocks include pigments, dyestuffs, imaging chemicals, agrochemicals, pharmaceuticals and plastics.^1^ In addition, alkylated nitroaromatics form the basis of many conventional explosives and energetic plasticisers, which are toxic and environmentally hazardous^2^ and which therefore require analysis^3^, ^4^, ^5^ and remediation methodologies.^6^ Methods for labelling alkylnitroaromatics are therefore of interest.

This paper describes the regiospecific benzylic deuteration of alkylnitroaromatics to provide labelled compounds for use in studies of the reactivity of alkyl‐2,4‐dinitrobenzenes under oxidising and nitrating conditions.^7^

To provide a general labelling method, we decided to approach the deuteration of this class of compounds via hydrogen isotope exchange methodology.^8^, ^9^

Isotopic exchange labelling of benzylic positions in conjunction with metal catalysts, sometimes with good specificity in particular cases, has been known for well over 50 years and continues to be of use.^10^, ^11^, ^12^, ^13^ Of current interest are nanoparticle catalysts that have been utilised very effectively and with good regiospecificity.^14^, ^15^ Alkali‐metal amide superbase catalysis has also been used recently.^16^

Unfortunately, the above approaches generally use hydrogen, deuterium or tritium gas as the initiator or isotope donor and are therefore inappropriate for the labelling of nitro‐compounds, which are easily reduced species. For the labelling of alkylnitroaromatics in the benzylic positions,^17^, ^18^, ^19^, ^20^, ^21^, ^22^ therefore, we decided to avoid reductive conditions and to utilise deuterium oxide as the donor. In addition, we exploited the differences in acidity between their aromatic and benzylic protons to provide the required labelling specificity.

**FIGURE 1:**
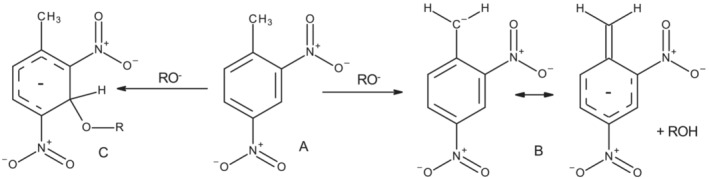
Potential species present when 2.4‐dinitrotoluene is treated with base

In alkylnitroaromatics, the benzylic protons are rendered particularly acidic by the presence of electron withdrawing nitro‐substituents,^23^ which can stabilise benzylic carbanions by resonance and inductive effects. However, studies of the interaction of 2,4,6‐trinitrotoluene (pKa 12 in DMSO/methanol) with ethoxide or hydroxide have shown the competing formation of an addition product.^24^ In this reaction, attack of the nucleophile at the position *meta* to the methyl group, or on the aromatic carbon bearing the methyl group, leads to anionic σ‐Meisenheimer species and renders the target methyl protons in these species unavailable for deuterium exchange. The analogous situation is summarised in Figure 1 for 2,4‐dinitrotoluene, a dinitro‐compound, which has been studied in most detail with respect to this behaviour.^25^ In this case it was shown that significant concentrations of potential Meisenheimer adducts (e.g., C) are avoided provided that the basic catalyst is an amine and the nitroaromatic is only weakly acidic. Under these conditions a small equilibrium concentration of the methylene carbanion (B) seems to be present. This renders the desired isotopic exchange likely, provided that a suitably acidic deuterium donor (e.g., D_2_O) is present. The striking blue colour of the reaction in the case of the dinitro‐compound was ascribed to the carbanion, though highly coloured complexes of 2,4,6‐trinitrotoluene with amines have also been proposed in which the interaction ranges from simple complex formation between the amine and the nitro‐substituents to the fully σ‐bonded Meisenheimer complexes.^26^, ^27^

**FIGURE 2:**
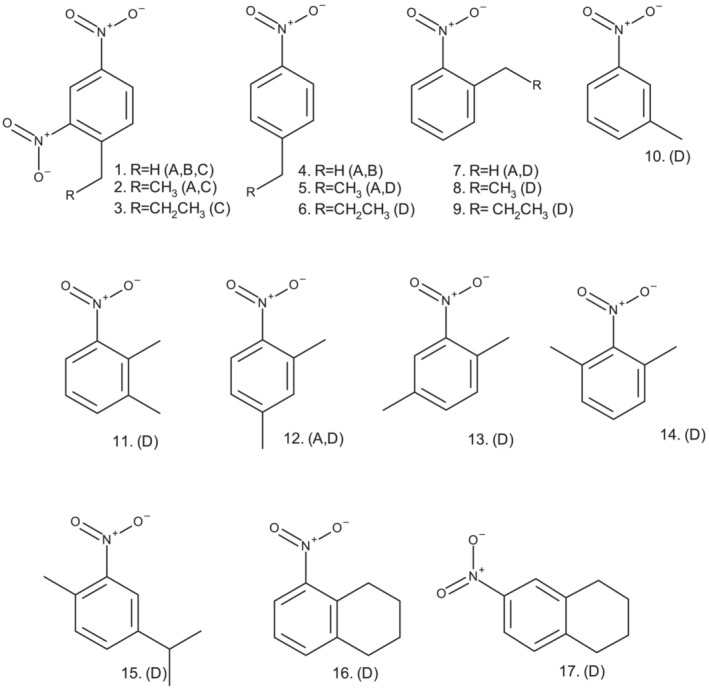
The substrates employed and their uses in the study. (A = synthetic preparations and labelling‐regiochemistry studies. B = time courses of deuteration (% deuterium vs. time), Figures 3 and 6. C = comparative deuteration rates of 1‐alkyl‐2,4‐dinitrobenzenes, Figure 5. D = comparison of the extent of deuteration of alkylmononitroaromatics under standard reaction conditions, Figure 7)

In the light of the above considerations, bearing in mind the inability of sodium hydroxide and deuterium oxide to facilitate deuteration in a previous investigation,^25^ we studied the efficacy of amine base‐catalysed isotope exchange between mononitro‐ and dinitro‐alkylaromatics and deuterium oxide using the substrates in Figure 2.

## 2 RESULTS AND DISCUSSION

We began our studies with 2,4‐dinitrotoluene (**1**). In this case, the benzylic protons are quite acidic (pKa 15.3) due to extensive resonance stabilisation of the benzylic carbanion by the two nitro‐substituents. Deuterium labelling of this compound had been noted previously^25^ when sodium hydroxide solutions were treated with deuterosulphuric acid. Interestingly, though, no deuterium exchange was noted without the addition of deuterosulphuric acid, implying that the benzylic carbanion itself was inert to exchange under treatment with a strong Bronsted base. However, we found that a simple tertiary amine base was efficient in catalysing the exchange of 2,4‐dinitrotoluene, provided that oxygen was excluded. If this was not the case, both the degree of deuteration and the yield were reduced.

**FIGURE 3:**
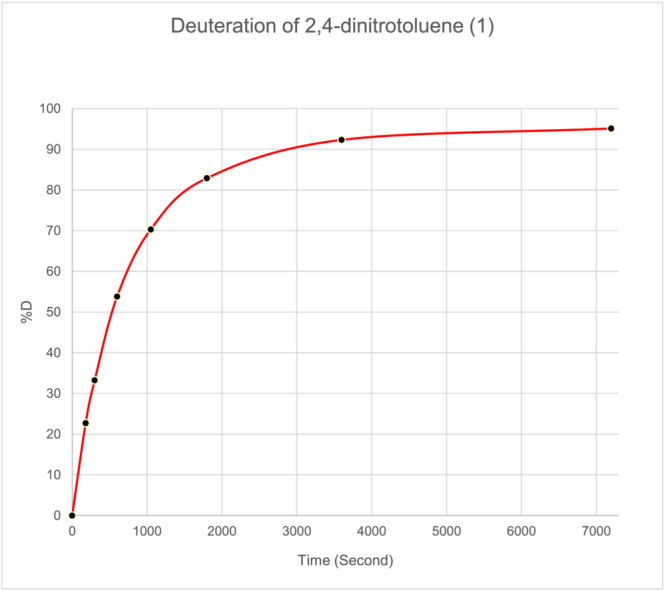
Labelling time‐course for 2,4‐dinitrotoluene in DMF/D_2_O at RT in the presence of triethylamine base (detailed conditions used are given in Section 4)

When carried out under nitrogen or argon, the labelling of 2,4‐dinitrotoluene proceeded smoothly at room temperature in tetrahydrofuran, and also in *N,N*‐dimethylformamide. Thus, [*methyl*‐D_3_]2,4‐dinitrotoluene was prepared easily, regiospecifically, and with high isotopic enrichment, under catalysis by triethylamine (pKa 10.75 in water) without any of the possible complications due to the formation of the Meisenheimer complex or of inhibition of the exchange by *stoichiometric* deprotonation to the carbanion. Figure 3 shows a typical time‐course for the labelling reaction.

**FIGURE 4:**
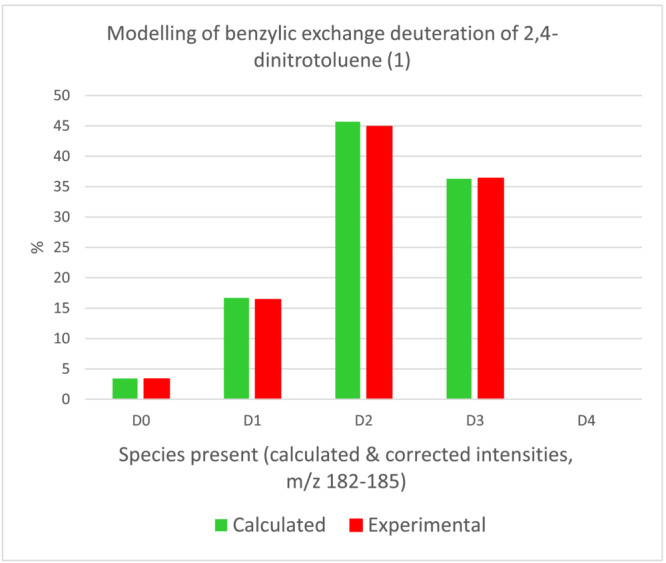
Statistical labelling behaviour of 2,4‐dinitrotoluene (**1**). Calculated distribution via LMSP^28^ (based on 69%D overall). Experimental raw MS data corrected via IsoPat2^29^ or NAIC^30^

Moreover, throughout, the labelling reaction followed the behaviour expected for a simple statistical H/D exchange^28^ (Figure 4 shows the data at 71.5% D; also see Data S1, section 1 for other examples of statistical labelling of alkylnitroaromatics).

However, the choice of the base catalyst did prove important, as the degree of deuteration achieved showed no simple correlation with base pKa (Data S1, section 2). In practice, both triethylamine and *N*‐methylpyrrolidine (pKas ca. 10.8) proved very effective with dinitro‐systems, provided, of course, the benzylic protons of the alkyl substituent were in an *ortho* or *para* relationship with the nitro‐groups.

**FIGURE 5:**
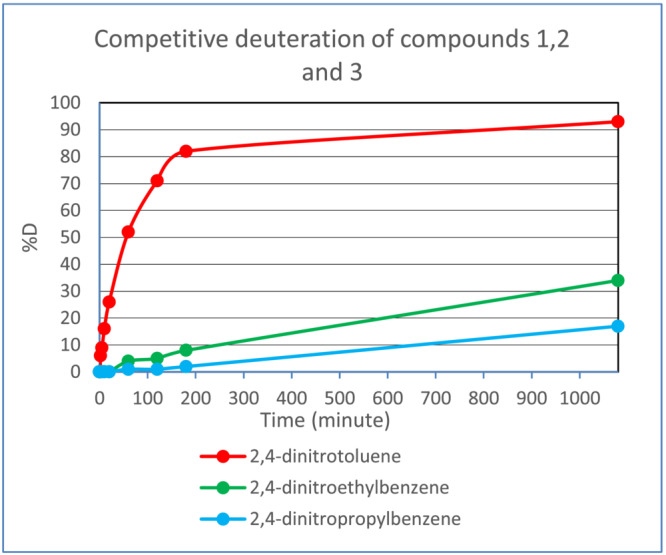
Effect of alkyl substituent on deuteration rate. Substrates were deuterated in THF/D_2_O at RT in the presence of triethylamine base (detailed conditions used are given in Section 4)

Figure 5 shows the relative rates of deuteration of three 2,4‐dinitroalkylbenzenes under competitive conditions (see Data S1, section 3 for a typical ion chromatogram). Clearly the rates for the higher alkyl substituents are slower. This could be due to the increased inductive (electron donating) effect of the extended alkyl group on the acidity of the benzylic protons (destabilisation of the carbanion): conversely it could be due to a steric effect, or to both these causes.

### 2.1 Labelling of mono‐nitro‐alkylaromatics

**FIGURE 6:**
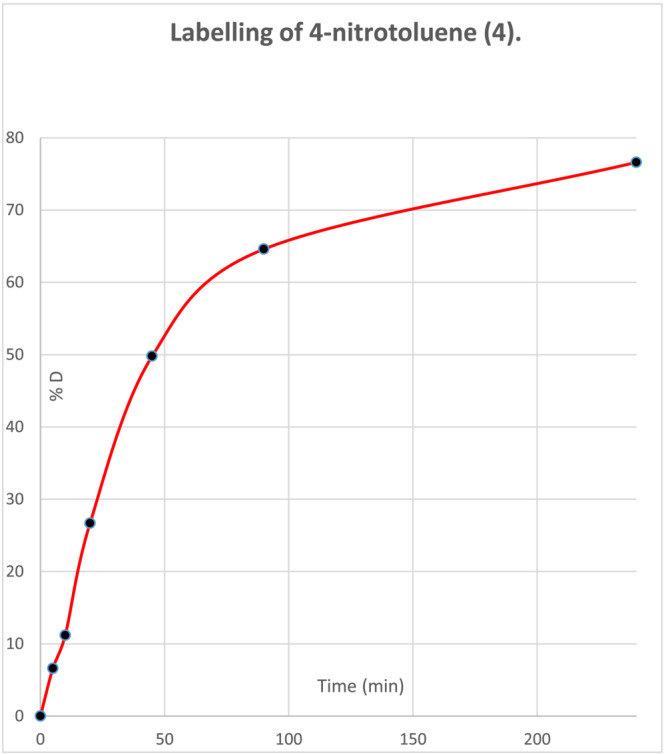
Labelling time course for 4‐nitrotoluene in DMF/D_2_O at 95°C in the presence of DBN base (detailed conditions are given in Section 4)

The benzylic protons of mono‐nitro compounds are much less acidic than those of dinitro‐systems (e.g., the pKa of 4‐nitrotoluene is ca. 22 in DMSO containing 5% water^23^). Nevertheless, mono‐nitro systems with the alkyl substituent *ortho* or *para* to the nitro group can still be labelled smoothly using this approach, provided that a stronger base (e.g., 1,5‐diazabicyclo[4.3.0]non‐5‐ene, DBN), a longer reaction time (overnight) and a higher temperature (95°C) are applied. Figure 6 shows a typical time course for 4‐nitrotoluene deuteration.

### 2.2 Factors affecting labelling ability

**FIGURE 7:**
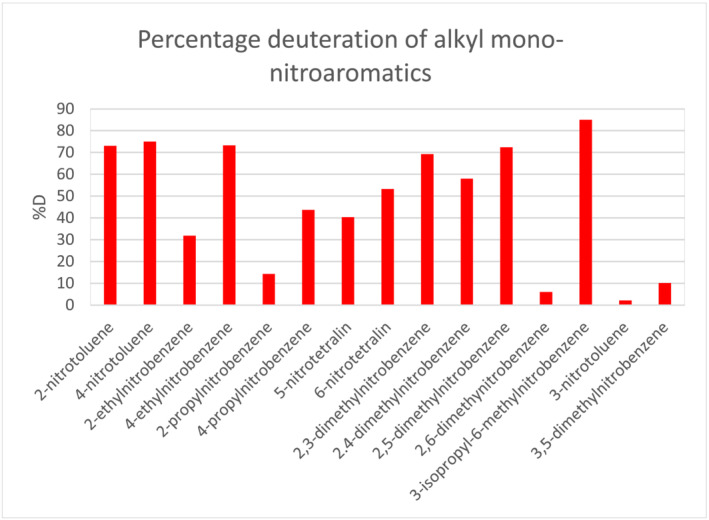
Deuteration of mono‐nitro alkylnitrobenzenes in DMF/D_2_O at 95°C in the presence of DBN base (detailed conditions are given in Section 4)

To determine the molecular factors affecting labelling propensity, the extent of deuteration of a range on alkylmononitrobenzenes was compared under identical conditions for a fixed reaction time (Figure 7). Competitive reactions were also carried out that showed the same rank ordering in the %D achieved. The resulting data suggest the following issues should be considered when applying the methodology.

Clearly, the orientation of the benzylic protons relative to the nitro groups is important. Labelling requires the nitro group to be *ortho* or *para* to the benzyl group, as expected if resonance stabilisation of the benzylic anion is a significant parameter facilitating the exchange.

Additionally, other sterioelectronic effects are significant in the exchange. In the alkyldinitroaromatics discussed earlier (Figure 5), the benzylic exchange at an ethyl substituent is less facile than that of a methyl group, whilst a propyl group reacts even more slowly. For the mono‐nitro substrates a similar pattern is seen for 2‐methyl, 2‐ethyl and 2‐propyl‐nitrobenzene (Figure 7). Moreover, the sluggish exchange of 2,6‐dimethylnitrobenzene compared with other dimethylnitrobenzenes also suggests the steric effect is significant in this case.

**FIGURE 8:**
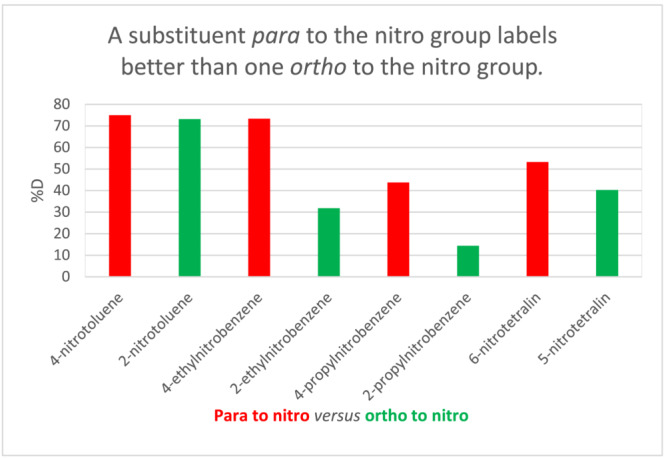
Effect of *para*‐ versus *ortho*‐nitro groups

Also of significance is the *ortho* versus *para* orientation of the alkylnitroaromatic. Figure 8 shows a comparison of such pairs. In all cases, the substrates in which the benzylic position is *ortho* to the nitro group labels less well than when the orientation is *para*. It is known that charge delocalisation by an *ortho*‐nitro substituent is less effective than for the corresponding *para* substituent^26^ making the *ortho* analogue less acidic by ca. 1.5 pK_a_ units.^23^

Bearing all the above behaviours in mind it seemed likely that steric inhibition of resonance was involved. Hence, the conformations of the substrates were examined using simple AM1 minimisation of the unsolvated ground states using Arguslab.4.0.1 or Spartan‐14 packages.

**FIGURE 9:**
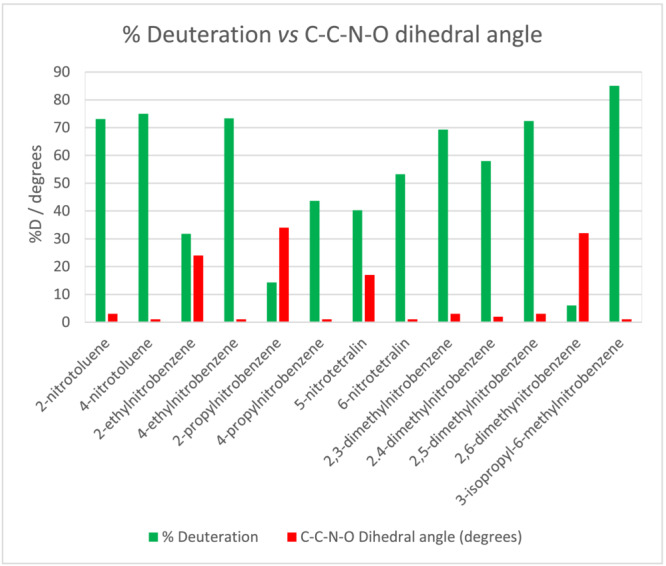
Effect of nitro‐group coplanarity

Figure 9 shows the relationship between the propensity for deuteration and the dihedral angle between the plane of the nitro group and the plane of the aromatic ring (see Data S1, section 4 for minimised conformations). Clearly the presence of bulky alkyl groups in an *ortho*‐orientation has the effect of twisting the nitro‐group out of plane and hence reducing the potential for resonance stabilisation of the benzylic anion. Studies using more sophisticated modelling of carbanion and transition state energies are indicated to confirm this interpretation and to identify the various factors leading to the reactive conformations or reactive species.

## 3 CONCLUSIONS

The benzylic protons of alkylnitroaromatics may be exchanged for deuterium from deuterium oxide under catalysis by amine bases. In the case of 2,4‐dinitro‐alkylaromatics, the exchange is facile in the presence of triethylamine, whereas mono‐nitro systems require a higher temperature and a stronger base, such as 1,5‐diazobicyclo[4.3.0]non‐5‐ene (DBN). Systems in which the benzylic protons are in an *ortho*‐orientation with respect to the nitro group label somewhat more slowly than those that are in a *para* relationship. The labelling reactions require an inert gas atmosphere, but otherwise are simple and high yielding with no obvious byproducts.

## 4 EXPERIMENTAL

### 4.1 Equipment

^1^H‐NMR spectra were recorded on Bruker spectrometers in CDCl_3_ at 500 MHz or 300 MHz for ^1^H and at 126 MHz or 100 MHz for ^13^C. ^2^H‐NMR spectra were recorded in C^1^HCl_3_ at 77 or 61 MHz. All spectra were analysed by Bruker TopSpin software. Raman spectra were measured on crystalline samples supported on glass microscope slides using a ThermoScientific DXR Raman Microscope at laser frequencies of either 532 or 780 nm. Infrared spectra were recorded for crystalline or neat liquid samples on a sapphire anvil using an Agilent Resolutions Pro spectrometer. GC‐MS analysis was performed using 1 μl injections of solutions at ca. 1 mg/ml in dichloromethane using an Agilent 6890N gas chromatograph coupled to a 5973 mass‐selective detector. The column was a Phenomenex ZB‐5MS, 30 m × 0.25 mm, used with a temperature profile: 50°C (held for 3 min), ramp at 10°C per minute to 250°C and held for 2 min. The carrier gas was helium at 1 ml/min.

### 4.2 Reagents

Substrates were purchased from Merck (Sigma‐Aldrich Company Ltd), The Old Brickyard, New Road, Gillingham, Dorset, SP8 4XT United Kingdom, or were prepared by nitration of the appropriate aromatic using nitric and sulphuric acids as specified below. Deuterium oxide (99.9 atom%D) and *N,N*‐dimethylformamide (DMF, <0.005% water content) were also obtained from Merck (address above). General solvents and reagents were obtained from regular chemical supply houses and were used as received.

#### 4.2.1 Determination of the percentage deuteration (isotopic abundance) of labelled compounds by mass spectrometry

The isotopic abundance of labelled nitroaromatics was determined by analysis of the integrated values of the whole GC‐MS peak of the labelled compound to avoid any inaccuracies arising from isotopic fractionation. The raw values from the molecular ion clusters of the labelled compound reported by the Agilent MassLynx software were then corrected for the presence of natural abundance isotopes using IsoPat^2^, ^29^ and NAIC^30^ software prior to calculation of the isotopic abundance.

#### 4.2.2 Preparation of deuteration substrates by nitration

Substrates not commercially available were prepared as below.

##### Nitration of ethyl‐4‐nitrobenzene to yield ethyl‐2,4‐dinitrobenzene

Ethyl 4‐nitrobenzene (5.0 g, 33.1 mmol) was pre‐washed with sodium sulphite solution (20 ml, 10% w/v) and water (2× 120 ml) before nitration by slow addition to a stirred nitrating mixture prepared from 70% nitric acid (12.8 g, 0.14 mol, d 1.42 g/ml) and concentrated sulphuric acid (19.2 g, 0.20 mol, d 1.84 g/ml) in an ice bath ensuring that the temperature did not rise above 20°C. The reaction was then left to stir at room temperature overnight. The resulting mixture was then transferred into a separating funnel and left standing for an hour to allow full separation of the phases. The organic layer was separated, washed with water (2×, 10 ml) and diluted with dichloromethane (15 ml). The dichloromethane solution was washed with sodium sulphite solution (10 ml, 5% w/v) and then stirred with potassium carbonate solution (10 ml, 5% w/v) for 30 min at ambient temperature. The organic layer was separated and dried overnight over anhydrous sodium sulphate. The solution was filtered and the solvent removed by evaporation under reduced pressure to leave ethyl 2,4‐dinitrobenzene as a yellow oil, 4.18 g, 64% ^1^H‐NMR (500 MHz, CDCl_3_) δ = 8.71 (d, *J* = 2.4 Hz, 1H), 8.37 (dd, *J*1 = 2.4 Hz, *J*2 = 8.5 Hz, 1H), 7.62 (d, *J* = 8.5 Hz, 1H), 3.02 (q, *J* = 7.5 Hz, 2H), 1.33 (t, *J* = 7.5 Hz, 3H) ppm. GC‐MS calculated for C_8_H_8_N_2_O_4_: 196.1, found 196.1.

##### Nitration of propylbenzene to yield propyl‐2‐nitrobenzene, propyl‐4‐nitrobenzene and propyl‐2,4‐dinitrobenzene

Propylbenzene (1.8 ml, 13 mmol) and concentrated sulphuric acid (3 ml) were stirred and cooled in ice and a mixture of concentrated nitric acid (0.5 ml) and concentrated sulphuric acid (1 ml) were added dropwise over 12 min. The reaction was allowed to attain room temperature with stirring for a further 21 min before being quenched with ice (25 g). Isolation of the products was carried out by partition of the reaction mixture between water (5 ml) and dichloromethane (5 ml). The dichloromethane extract was then washed with water (four portions of 5 ml) and dried. Removal of the dichloromethane yielded a mixture containing 2‐propynitrobenzene, 4‐propylnitrobenzne and 1‐propyl‐2,4‐dinitrobenzene. A portion of this mixture was separated into its components by column chromatography using a silicagel column eluted with hexane containing increasing quantities of dichloromethane (from 10% to 40% v/v). The products were analysed by GC‐MS and ^1^H‐NMR.

##### 2‐Propylnitrobenzene (**9**)

*m/z* 165 (M+, 2%), 148 (100%), 130, 120, 115, 106, 91, 77, 65, 57, 51; δ ^1^H‐NMR (CDCl_3_), 0.99 (t, *J* = 7.5 Hz, 3H), 1.65 (hexuplet, *J* = 7.5 Hz, 2H), 2.88 (t, *J* = 7.5 Hz, 2H), ca. 7.33 and 7.34 (overlapping triplet and doublet, *J* = ca. 8 Hz, 2H), 7.50 (dd, *J* = ca. 8 Hz, 1.2 Hz, 1H), 7.86 (d, *J* = ca. 8.1 Hz, 1H) ppm.

##### 4‐Propylnitrobenzene (**6**)

*m/z* 165 (M+, 84%), 148, 136, 119, 106, 91, 78, 63, 51; ^1^H‐NMR (500 MHz, CDCl_3_), δ 0.93 (t, *J* = ca. 7.3 Hz, 3H), 1.68 (cm, 2H), 2.70 (t, *J* = ca. 7.4 Hz, 2H), 7.32 (d, *J* = 8.7 Hz, 2H), 8.14 (d, *J* = 8.7 Hz, 2H), ppm.

##### 1‐Propyl‐2,4‐dinitrobenzene (**3**)

*m/z* 210 (M+, 100%), 193 (86%), 147 (71%). δ 1H‐NMR (CDCl3), 1.00 (t, *J* = ca. 7.3 Hz, 3H), 1.73 (cm, *J* = ca. 7.6 2H), 2.97 (t, *J* = ca. 7.8 Hz, 2H), 7.58 (d, *J* = ca. 8.5 Hz, 1H), 8.4 (dd, *J* = ca. 8.5, 2.3 Hz, 1H), 8.7 (d, *J* = ca. 2.3 Hz, 1H) ppm.

##### Nitration of tetralin to yield 5‐nitrotetralin and 6‐nitrotetralin

Tetralin (1,2,3,4‐tetrahydronaphthalene, 2 mmol) was dissolved in dichloromethane (10 ml) and concentrated sulphuric acid (324 μl) was added. Then two portions of nitric acid (81 μl each time) were added over 5 min. After stirring for a further 2 h, the reaction mixture was neutralised by the addition of saturated sodium bicarbonate solution. After discarding the aqueous layer the dichloromethane layer was washed twice with water (10 ml), dried and evaporated. NMR analysis showed the product to be a mixture of 5‐ and 6‐nitrotetralin. The products were partially separated using a column chromatography (silicagel, increasing quantities of dichloromethane in hexane from 10% to 50% v/v). The two products were then further purified by preparative thin layer chromatography (silicagel, 50% dichloromethane in hexane) and stripped from the stationary phase using 50% ethyl acetate in dichloromethane before characterisation by GC‐MS and ^1^H‐NMR.

##### 1,2,3,4‐Tetrahydro‐5‐nitronaphthalene (5‐nitrotetralin, **16**)

*m/z* 177, 159, 130 (100%), 117, 115, 103, 91, 77, 63, 51; δ ^1^H‐NMR (CDCl_3_), 1.8 (broad cm, 4H), 2.88 (broad, 2H), 2.96 (broad, 2H), 7.20 (t, *J* = ca. 7.9 Hz, 1H), 7.29 (d, *J* = ca. 7.9 Hz, 1H), 7.65 (d, *J* = ca. 8.0 Hz, 1H) ppm.

##### 1,2,3,4‐Tetrahydro‐6‐nitronaphthalene (6‐nitrotetralin, **17**)

*m/z* 177 (100%), 160, 149, 131, 115, 91, 77, 63, 51; δ ^1^H‐NMR (CDCl_3_) 1.81 (cm, 4H), 2.86 (broad, 4H), 7.19 (d, *J* = ca. 8.4 Hz, 1H), 7.92 (dd, *J* = ca. 8.4 Hz, 2.2 Hz, 1H), 7.94 (d, *J* = ca. 2.2 Hz, 1H) ppm.

### 4.3 Labelling reactions

#### 4.3.1 Labelling time‐course for 2,4‐dinitrotoluene

2,4‐Dinitrotoluene (15 mg) was dissolved in dry *N,N*‐dimethylformamide (500 μl) and D_2_O (100 μl) added. The tube was placed under nitrogen and then triethylamine (100 μl) added via a syringe. Aliquots (ca. 50 μl) were removed by syringe at the indicated times, neutralised with 37% DCl in D_2_O (50 μl), partitioned between dichloromethane (2 ml) and water (1 ml) and the dichloromethane layer washed with water (1 ml), dried and analysed by GC‐MS.

#### 4.3.2 [Methyl‐D3]2,4‐dinitrotoluene

2,4‐Dinitrotoluene (0.1 mmol) was dissolved in dry tetrahydrofuran (0.5 ml) and D_2_O (100 μl) added. The reaction tube was sealed with a septum and thoroughly flushed with nitrogen. Triethylamine (100 μl) was then injected via a syringe, resulting in the deep blue colour of the benzylic anion. This colour changed slowly to green over the subsequent 18 h. After this period 37% DCl in D_2_O (100 μl) was injected to terminate the reaction. Extraction of the labelled product was carried out by partition between dichloromethane (10 ml) and water (10 ml). The organic extracts were washed with water (4×, 10 ml) and the dichloromethane layer separated, dried & evaporated to yield straw‐coloured crystals of [methyl‐D_3_]2,4‐dinitrotoluene, 95%, 95.5%D (calculated for three atoms, 98% of theoretical labelling), 98% pure by GC‐MS: *m/z* 185, 167 (100%), 121, 109, 92, 81, 64, 53; δ (^1^H‐NMR, CDCl_3_, 500 MHz) 2.63 (cm, <0.02H), 7.53 (d, *J* = 8.4 Hz, 1H), 8.29 (dd, *J* = 2.3, 8.4 Hz, 1H), 8.73, (d, *J* = 2.3 Hz, 1H) ppm; δ (^13^C‐NMR, CDCl_3_, 126 MHz) 20.1 (cm, *J* = ca. 20 Hz), 120.2, 126.9, 134.0, 140.6, 146.6, 149.1 ppm; δ (^2^H‐NMR, CHCl_3_, 61.4 MHz) 2.69 (s, no other resonances) ppm; ν_max_ 3105, 2866, 2741, 2362, 1599, 1516, 1344, 1266, 1214, 1150, 1075, 1032, 913, 836, 769, 723, 623 cm^−1^; Raman (glass, 780 nm excitation) 3088, 2141, 1613, 1545, 1527, 1358/1348 (strong close doublet), 1313, 1219, 1153, 1135, 1051, 842, 455, 160 cm^−1^.

#### 4.3.3 1‐[Ethyl‐1′‐d]ethyl‐2,4‐dinitrobenzene

1‐Ethyl‐2,4‐dinitrobenzene (1.25 g, 6.4 mmol) was dissolved in DMF (42 ml) and deuterium oxide (8.3 ml) in a round‐bottomed flask sealed under nitrogen via a rubber septum. Triethylamine (8 ml, 5.81 g, 57.4 mmol) was then added via a syringe and the solution was stirred at ambient temperature for 24 h. The reaction was then neutralised with 37% DCl in D_2_O and partitioned between water (60 ml) and dichloromethane (60 ml). The organic layer was washed with water (3×, 60 ml) and dried overnight over anhydrous sodium sulphate. Filtration and evaporation of the solvent gave 1‐[ethyl‐1′‐d]ethyl‐2,4‐dinitrobenzene, 53% as a yellow oil. ^1^H‐NMR (300 MHz, CDCl_3_) δ: 1.33 (bs, 3H), 3.05 (cm, trace, residual partially labelled methylene), 7.61 (d, *J* = 8.5 Hz, 1H, H6), 8.37 (dd, *J* = 2.5 & 8.6 Hz, 1H), 8.74 (d, *J* = 2.3 Hz, 1H), *m/z* 198, 180, 163, 134, 105, 92, 78, 63, C_8_H_6_D_2_N_2_O_4_: 198.1 found 198.1, The percentages of dideuterated, monodeuterated and unlabelled compound were calculated^29^, ^30^ from the MS molecular ion intensities to be 81%, 18%, and 1%, respectively.

#### 4.3.4 Labelling time course for 4‐nitrotoluene

4‐Nitrotoluene (27.5 mg) was dissolved in dry *N,N*‐dimethylformamide (1 ml) in a thick‐walled septum tube containing a stirrer bar and D_2_O (200 μl) added. The tube was capped, flushed with nitrogen and then 1,5‐diazabicyclo[4.3.0]non‐5‐ene, DBN, (400 μl) added via a syringe. The tube was then placed in a heating block and stirred at 95°C. Aliquots (ca. 100 μl) were removed by syringe at the indicated times, neutralised with 37% DCl in D_2_O (100 μl), partitioned between dichloromethane (2 ml) and water (1 ml) and the dichloromethane layer washed with water (1 ml), dried and analysed by GC‐MS.

#### 4.3.5 [Methyl‐d]4‐nitrotoluene

4‐Nitrotoluene (69 mg, 0.5 mmol) was dissolved in a mixture of dry *N,N*‐dimethylformamide (2.0 ml) and deuterium oxide (1.0 ml) and the mixture sealed under argon. DBN (1,5‐diazobicyclo[4.3.0]non‐5‐ene, 1.0 ml) was then added via syringe and the reaction tube heated at 95 degrees for 18 h. After cooling, the labelling reaction was terminated by the addition of 37% DCl in D_2_O (1 ml). The reaction mixture was then partitioned between dichloromethane (10 ml) and 4N hydrochloric acid (6 ml) and the dichloromethane layer separated and washed four times with water (8 ml each time) before being dried over anhydrous magnesium sulphate, filtered, and the filtrate evaporated at 70 degrees to constant weight to yield [methyl‐d]4‐nitrotoluene (62 mg, 88%), 78.82 ± 0.24% D (calculated for three atoms), *m/z* (largest peaks in isotope clusters) 140, 124, 110, 94, 79, 67, 51; δ ^1^H‐NMR (500 MHz, CDCl_3_) δ 2.43 (cm, ca. 0.6H), 7.31 (d, *J* = 8.3 Hz, 2H), 8.11 (d, *J* = 8.3 Hz, 2H) ppm; δ ^2^H‐NMR (61.4 MHz, CHCl_3_) 2.32 (s. CD_3_) 2.36 (s, CHD_2_) ppm; δ (^13^C‐NMR, CDCl_3_, 126 MHz) 20.7 (cm, *J* = ca. 20 Hz), 123.5, 129.8, 145.9 (cm), 146.2 ppm; ν_max_ (sapphire) 3107, 3082, 2935, 2841, 2450, 1944. 1597 (s), 1505 (s), 1340 (s). 1178, 1106, 903, 856, 812, 766, 722 (s) cm^−1^; Raman (glass, 780 nm excitation): 3078, 2940, 2670, 2133, 1604, 1600, 1513, 1508, 1113, 1103, 860, 633, 350, 270 cm^−1^.

#### 4.3.6 [Methyl‐d]2‐nitrotoluene

2‐Nitrotoluene (45 mg, 0.33 mmol) was dissolved in a mixture of dry *N,N*‐dimethylformamide (1.0 ml) and deuterium oxide (0.6 ml) and the mixture sealed under argon. DBN (1,5‐diazobicyclo[4.3.0]non‐5‐ene, 0.4 ml) was then added and the tube heated at 95 degrees for 18 h. The labelling was terminated by the addition of 37%. DCl in D_2_O (0.5 ml). The reaction mixture was then partitioned between dichloromethane (10 ml) and 4N hydrochloric acid (7 ml) and the dichloromethane layer separated and washed four times with water (10 ml each time) before being dried over anhydrous magnesium sulphate, filtered, and the filtrate evaporated at 70 degrees to constant weight to yield [methyl‐d]2‐nitrotoluene (38 mg, 83%), 78.2% D (calculated for three atoms), *m/z* (largest peaks in isotope clusters) 140, 122, 108, 94, 79, 66, 51; δ ^1^H‐NMR (500 MHz, CDCl_3_) 2.56 (cm, ca. 0.8H), 7.34 (Overlapping t and d, *J* = ca. 7.4 and ca. 8.6 Hz, 2H), 7.49 (t, *J* = 7.5 Hz, 1H), 7.97 (d, *J* = 8.3 Hz, 1H) ppm; δ (^13^C‐NMR, CDCl_3_, 126 MHz) 20.0 (cm, *J* = ca. 20 Hz), 124.6, 126.9, 132.7, 132.9, 133.5 (cm), 149.4 ppm; ν_max_ (sapphire) 3068, 2928, 2857, 1679, 1612, 1576, 1518 (s), 1344 (s), 1307, 1275, 1088, 1046, 864, 786, 718 (s), 663 cm^−1^.

#### 4.3.7 4‐[Methylene‐d]ethylnitrobenzene

4‐Ethylnitrobenzene (51 mg 0.34 mmol) was dissolved in a mixture of dry *N,N*‐dimethylformamide (1.0 ml) and deuterium oxide (0.6 ml) and the mixture sealed under argon. DBN (1,5‐diazobicyclo[4.3.0]non‐5‐ene, 0.4 ml) was then added via a syringe and the tube heated at 95 degrees for 18 h. The labelling was terminated by the addition of 37%. DCl in D_2_O (0.5 ml). The reaction mixture was then partitioned between dichloromethane (10 ml) and 4N hydrochloric acid (7 ml) and the dichloromethane layer separated and washed four times with water (10 ml each time) before being dried over anhydrous magnesium sulphate, filtered, and the solvent evaporated at 70 degrees to constant weight to yield [methylene‐d]4‐ethylnitrobenzene (45 mg, 87%), 74.07 ± 0.04%D (calculated for two atoms), *m/z* (largest peaks in clusters) 153, 138, 123, 107, 91, 78, 63, 51; δ ^1^H‐NMR (500 MHz, CDCl_3_) 1.23–1.26 m, 3H), 2.75 (cm, ca. 0.46H), 7.34 (d, *J* = 8.6 Hz, 2H), 8.14 (d, *J* = 8.6 Hz, 2H); δ ^2^H‐NMR (61.4 MHz, CHCl_3_) 2.74 (s) ppm; δ (^13^C‐NMR, CDCl_3_, 126 MHz) 14.89, 14.96, 15.03, 24.5 (cm, *J* = ca. 20 Hz), 123.6, 128.6, 146.3, 152.0 ppm; ν_max_ (sapphire), 2969 (s), 2933, 1601 (s), 1512 (s), 1456, 1342 (s), 1180, 1110, 1016, 855, 743, 697, 631 cm^−1^.

#### 4.3.8 2,4‐[2,4‐Methyl‐d]dimethylnitrobenzene

2,4‐Dimethylnitrobenzene (51 mg, 0.34 mmol) was dissolved in a mixture of dry *N,N*‐dimethylformamide (1.0 ml) and deuterium oxide (0.6 ml) and the mixture placed under argon as above. DBN (1,5‐diazobicyclo[4.3.0]non‐5‐ene, 0.4 ml) was then added and the tube heated at 95 degrees for 18 h. The labelling was terminated by the addition of 37%. DCl in D2O (0.5 ml). The reaction mixture was then partitioned between dichloromethane (10 ml) and 4N hydrochloric acid (7 ml) and the dichloromethane layer separated and washed four times with water (10 ml each time) before being dried over anhydrous magnesium sulphate, filtered and the solvent evaporated at 70 degrees to constant weight to yield [dimethyl‐d]2,4‐dimethylnitrobenzene (46 mg, 88%), 51.84 ± 0.11%D (calculated for six atoms). *m/z* (largest peaks in isotope clusters) 153, 138, 123, 107, 91, 78, 63, 51; δ ^1^H‐NMR (500 MHz, CDCl_3_) 2.38 (cm, ca. 1.9H), 2.56 (cm, ca. 1H), 7.12 (d, *J* = ca. 8.4 Hz, 1H), 7.13 (s, 1H), 7.91 (d, *J* = ca. 8.4 Hz, 1H) ppm; δ ^2^H‐NMR (61.4 MHz, CHCl_3_) 2.37, 2.40, 2.44, 2.56, 2.60, 2.63 ppm in intensity ratios 33:41:15:100:59:20; δ (^13^C‐NMR, CDCl_3_, 126 MHz) 19.9‐21.3 (cm, *J* = ca. 20 Hz), 124.9, 127.5, 133.3, 133.7 (cm), 144.1 (cm)147.0 ppm; ν_max_ (sapphire) 3040, 2930, 2852, 1612, 1586, 1511 (s), 1340 (s), 1279, 1155, 1083, 1043, 938, 833, 739, 630 cm^−1^.

(Further spectroscopic data for all the above labelled compounds are provided in Data S1, sections 5 and 6).

### 4.4 Comparative deuteration of 2,4‐dinitrotoluene, ethyl‐2,4‐dinitrobenzene and propyl‐2,4‐dinitrobenzene

The three substrates (0.1 mmol each) in tetrahydrofuran (1.0 ml) and deuterium oxide (200 μl) were placed in a septum‐capped tube and sealed under nitrogen. Triethylamine (200 μl) was then added via a polypropylene syringe and the tube shaken. Samples (ca. 100 μl) were removed via a gas‐tight syringe at 1, 2, 5, 10, 20, 60, 120, 180 and 1100 min and added to 37% deuterochloric acid in deuterium oxide (100 μl). The resulting mixture was partitioned between dichloromethane (5 ml) and water (10 ml), the dichloromethane layer separated, washed with water (2 x 5 ml), and dried. An aliquot of the dichloromethane extract was then analysed by GC‐MS to determine the percentage deuteration of each of the substrates at each time point.

### 4.5 Protocol for comparison of the extent of deuteration of alkylmononitroaromatics

The substrates (0.1 mmol) in *N,N*‐dimethylformamide (500 μl) and deuterium oxide (100 μl) were placed in thick‐walled septum‐capped pressure tubes and sealed under nitrogen. Next, 1,5‐diazabicyclo[4.3.0]non‐5‐ene, DBN, (200 μl), was injected into each tube via a syringe. The reaction tubes were heated at 94°C for 4 h using a heating block. The reactions were run under conditions where equilibrium had not been achieved so that comparisons of the degree of deuteration could be made. After cooling, the tubes were decapped and 37% deuterochloric acid in deuterium oxide (100 μl) was added. The resulting solution was partitioned between water (5 ml) and deuterochloroform (1.5 ml). The deuterochloroform phase was then washed with water (4×, 5 ml), separated and dried, before confirmation of identity by TLC and GC retention time and determination of the percentage deuteration by GC‐MS.

*[methyl‐d ]2‐Nitrotoluene: m/z* (73.1%D, Major peak in isotope clusters) 140, 122, 108, 94, 79, 66, 51.

*[methyl* ‐*d ]4‐Nitrotoluene: m/z* (75.0%D, Major peak in isotope clusters) 140/139, 110/109, 94/93, 82, 67.

*2,3‐[2,3‐methyl* ‐*d ]Dimethylnitrobenzene: m/z* (69.3%D, Major peak in isotope clusters) 153, 136, 107, 105, 80, 78, 65, 63, 51.

*2,5‐[2,5‐methyl* ‐*d ]Dimethylnitrobenzene: m/z* (72.4%D, Major peak in isotope clusters) 153, 136, 107, 93, 80, 78, 67, 51.

*1‐[ethyl‐1′* ‐*d ]Ethyl‐2‐nitrobenzene*: *m/z* (31.8%D, Major peak in isotope clusters) 153/152 (weak), 135, 118, 107, 104, 93, 78, 66, 51.

*1‐[ethyl‐1′* ‐*d ]Ethyl‐4‐nitrobenzene: m/z* (73.3%D, Major peak in isotope clusters) 153, 138, 123, 107, 80, 63, 51.

*1‐[propyl‐1′* ‐*d ]Propyl‐2‐nitrobenzene: m/z* (14.3%D, Major peak in isotope clusters) No detectable molecular ion, 149/148, 130, 120, 114, 106, 91, 78, 65, 63, 57, 51; δ ^1^H‐NMR (500 MHz, CDCl_3_) 0.99 (t, 3H),1.67 (hexuplet, 2H), 2.86 (t, ca. 1.6H), 7.33 (t, 2H), 7.34 (d, 1H), 7.5 (t, 1H), 7.86 (d, 1H) ppm.

*1‐[propyl‐1′* ‐*d ]Propyl‐4‐nitrobenzene: m/z* (43.7%D, Major peak in isotope clusters) 166/165, 150, 137, 120, 116, 108, 92/91, 79, 63, 51.

*3‐Isopropyl‐6‐[methyl* ‐*d ]methylnitrobenzene: m/z* (85.0%D, Major peak in isotope clusters) 181, 166, 148, 135, 119, 106, 92, 78, 66, 51.

*1,2,3,4‐[4*‐*d]Tetrahydro‐5‐nitronaphthalene ([4*‐*d]5‐nitrotetralin): m/z* (40.3%D, Major peak in isotope clusters) 179, 178, 159, 130, 116, 104, 91, 78, 63, 51.

*1,2,3,4‐[1* ‐*d ]Tetrahydro‐6‐nitronaphthalene ([1*‐*d ]6‐Nitrotetralin): m/z* (53.2%D, Major peak in isotope clusters) 179, 178, 162, 149, 132, 117, 103, 92, 78, 63, 51.

## Supporting information

Data S1: Section 1: Experimental and calculated isotope distribution functions for various substrates.
Section 2: Deuteration of 2,4‐dinitrotoluene using various bases as catalyst.
Section 3: Typical GC‐MS of comparative deuteration of methyl‐, ethyl, and propyl‐2,4‐dinitrobenzenes.
Section 4: Ground state structures of various alkylnitrobenzenes from Argus Lab Am1 minimisation and energy diagrams from Spartan 14.
Section 5: Deuterium NMR data.
Section 6: Carbon‐13 NMR data.

## Data Availability

The data that support the findings of this study are available from the corresponding author upon reasonable request.
