# Supplementary material for: Benzylic deuteration of alkylnitroaromatics via amine‐base catalysed exchange with deuterium oxide
Source: J Labelled Comp Radiopharm. 2022 Dec 16;66(1):11–21. doi: 10.1002/jlcr.4008 (PMC10107807; doi:10.1002/jlcr.4008)
Supplement: Supplementary file 1 — Data S1: Section 1: Experimental and calculated isotope distribution functions for various substrates. Section 2: Deuteration of 2,4‐dinitrotoluene using various bases as catalyst. Section 3: Typical GC‐MS of comparative deuteration of methyl‐, ethyl, and propyl‐2,4‐dinitrobenzenes. Section 4: Ground state structures of various alkylnitrobenzenes from Argus Lab Am1 minimisation and energy diagrams from Spartan 14. Section 5: Deuterium NMR data. Section 6: Carbon‐13 NMR data. [file JLCR-66-11-s001.docx]

**Supplementary data**

**Section 1**: Experimental and calculated isotope distribution functions for various substrates.

**Section 2**: Deuteration of 2,4-dinitrotoluene using various bases as catalyst.

**Section 3**: Typical GC-MS of comparative deuteration of methyl-, ethyl, and propyl-2,4-dinitrobenzenes.

**Section 4**: Ground state structures of various alkylnitrobenzenes from Argus Lab Am1 minimisation and energy diagrams from Spartan 14.

**Section 5:** Deuterium NMR data.

**Section 6:** Carbon-13 NMR data.

**Supplementary data.**

**Section 1**

The isotope distribution function (IDF) for an MS ion can be calculated after correction of the raw MS data by programs (NAIC*, IsoPat^2^) which correct for the presence of heavy isotopes at natural abundance. After such correction, simple statistics results in:

IDF = (A+B)^n^ x (C+D)^o^ x (E+F)^p^ x (G+H)^q^…..etc

Where:

A= fraction of light isotope at first site

B= fraction of heavy isotope at first site

n = number of possible atoms at that site

C= fraction of light isotope at second site

D= fraction of heavy isotope at second site

o= number of possible atoms at that site

E=.etc

The expected MS peak distribution is then the sum of the terms in the expansion of the complete expression, bearing in mind the degree of substitution on the mass. (i.e., collecting up terms with no heavy isotope, ones with one heavy isotope, ones with two heavy isotopes, three heavy isotopes... etc).  The calculation of the IDF is facilitated by an MS Windows program (see “A statistical approach to the analysis of MS ions of compounds labelled with ^2^H, ^3^H, ^13^C and ^14^C”. W.J.S. Lockley, R. Sherhod, *J Label Compd Radiopharm*, 2008, 51, 258) which is available from the corresponding author.

Since the above expression assumes statistical distribution of isotopes between the possible exchange sites, a simple corrected mass spectrum in conjunction with IDF analysis can provide information about the types of exchange sites in the molecule in terms of their degree of deuteration and the number of exchangeable protons at each site. E.g. a phenyl group could show good fits to one exchangeable site (*para*), two exchangeable sites (*ortho* or *meta*), three exchangeable sites (*para* plus *ortho* or meta), four exchangeable sites (*ortho* plus *meta*) or five exchangeable sites with different degrees of deuteration between the *ortho*, *para* and *meta* sites.

Below is an example of LMSP analysis of a phenyl group deuterated symmetrically but differently in the para (71%D), meta (51%D) and ortho (22%D) positions.


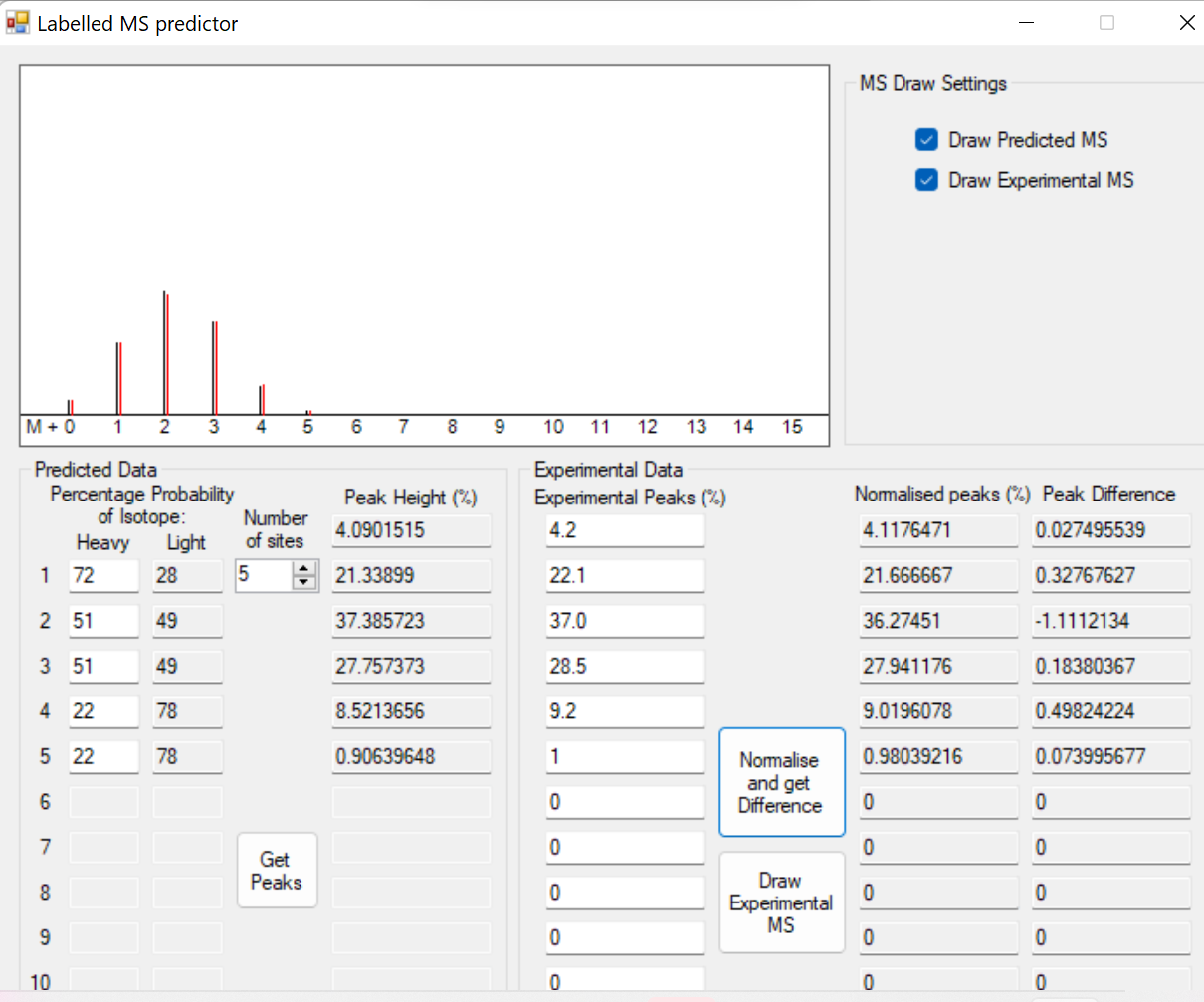


With respect to correction of the MS for heavy isotopes at natural abundance using IsoPat2 or NAIC , correction for deuterium is unproblematic, but other isotopes , e.g. carbon-14 and tritium are more complicated. See D J Schenk, W J S Lockley, C S Elmore, D Hesk, D Roberts*, J Labelled Comp Radiopharm*, 2016 Apr;59(4):136-46. doi: 10.1002/jlcr.3370. Epub 2016 Feb 24.

**Typical calculated and experimental data for isotope distribution for the compounds studied at the specified extent of deuteration**

**
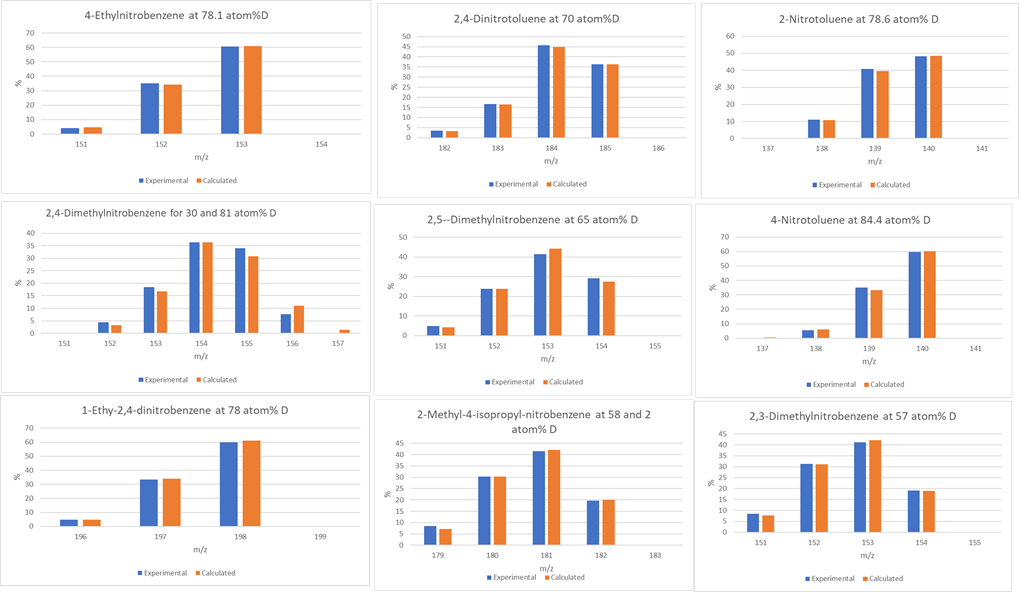
**

**Section 2**

**Deuteration of 2,4-dinitrotoluene by basic catalysts of varying pKa**

| **Base** | **pKa** | **Deuteration (%)** |
| --- | --- | --- |
| *N-*methylpyrrolidine | 10.5 | 96 |
| *N,N-*dimethylaniline | 4.85 | 0.3 |
| *N,N*-dimethylpiperazine | 8.4 | 56.4 |
| *N*-methylmorpholin | 7.4 | 11.3 |
| 1,8-Bis(dimethylamino)naphthalene [Proton Sponge] | 12.1 | 0.2 |
| 1,5-Diazabicyclo(4.3.0)non-5-ene [DBN] | 13.5 | 78.4 |
| Triethylamine | 10.7 | 96 |
| *N,N, N’,N’*-tetramethyl-hexamethylene-1,6-diamine | 9.33 | 71.4 |
| 1,4-Diazabicyclo[2.2.2]octane [DABCO] | 8.7 | 85.9 |
| 4-Dimethylaminopyridine [DMAP] | 9.7 | 36 |
| N,N, N’,N’-tetramethylethylenediamine | 9 | 89 |
| N,N, N’,N’-tetramethylmethylenediamine | 7.5 | 88.4 |
| Quinuclidine | 12.1 | 90.2 |
| *N,N*-dimethylbenzylamine | 8.91 | 79.9 |

From this data there is no clear correlation between pKa and catalytic ability.

(It is noteworthy that under this comparison protocol, six of the fourteen data points were at 80%D or greater and hence would not show much differentiation. However, deleting all the data above 80% only leads to a correlation coefficient change from 0.1139 to 0.1511. Nevertheless, it remains possible that the correlation might have been clearer if all the reactions had been terminated at earlier time points).

**Section 3**

**Typical GC-MS analysis of competitive deuteration of methyl-, ethyl- and propyl-2,4-dinitrobenzene**


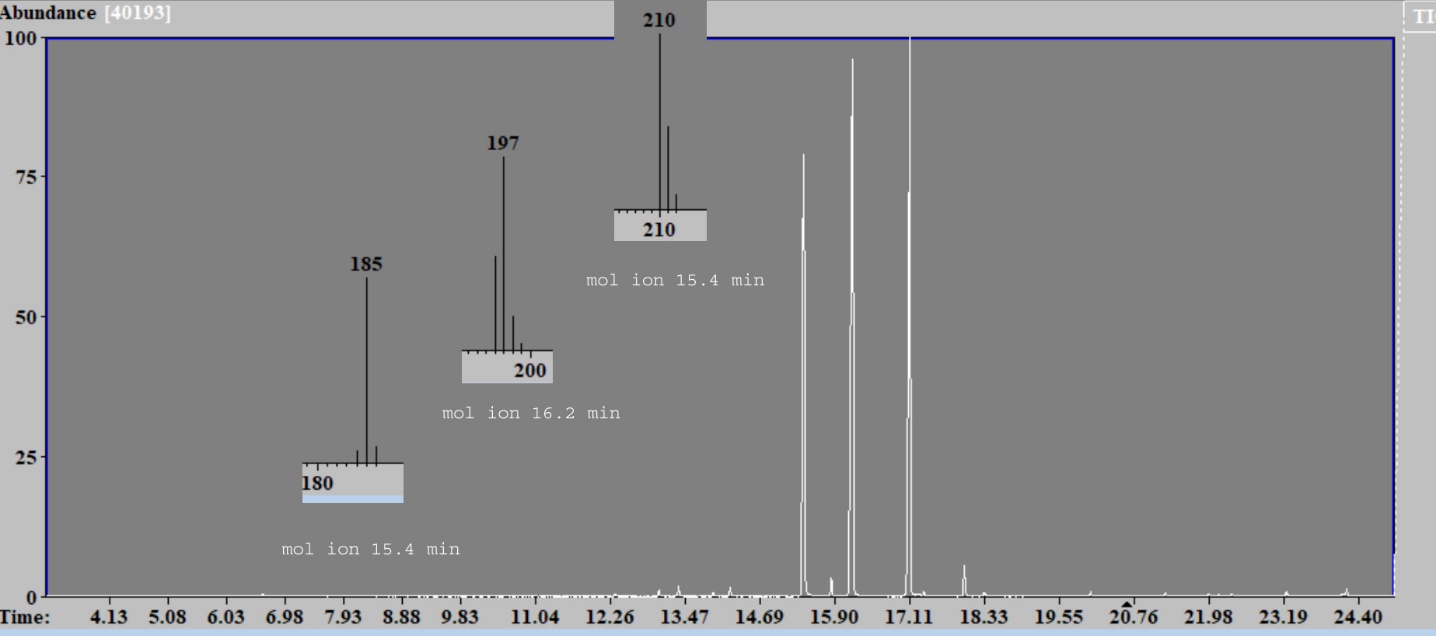


At this time point deuteration is essentially complete for Me, partial for Et and small for Pr.

**Section 4**

**Argus Lab minimised ground state conformations of the substrates studied**

2-nitrotoluene, 4-nitrotoluene, 2-ethylnitrobenzene

4-ethylnitrobenzene, 5-nitrotetralin, 6-nitrotetralin

4-propylnitrobenzene, 2-propylnitrobenzene, 2,3-dimethylnitrobenzene

2,4-dimethylnitrobenzene, 2,5-dimethylnitrobenzene,2,6-dimethylnitrobenzene

3-isopropyl-6-methylnitrobenzene, 3-methylnitrobenzene, 3,5-dimethylnitrobenzene

**Comparison energy diagrams from Spartan 14 analysis of nitro group bond rotation for groups of nitroaromatics.**

**
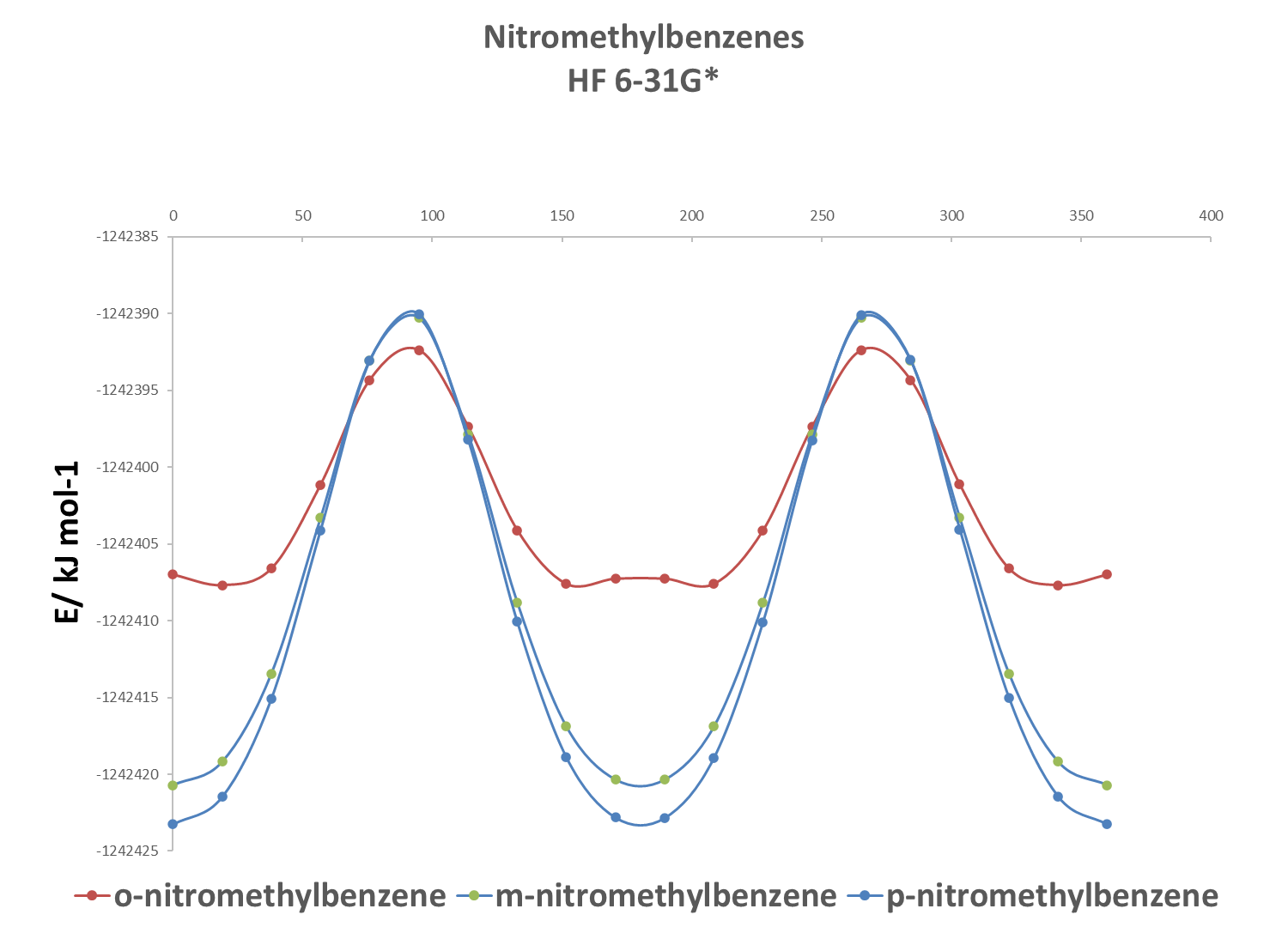
**

**
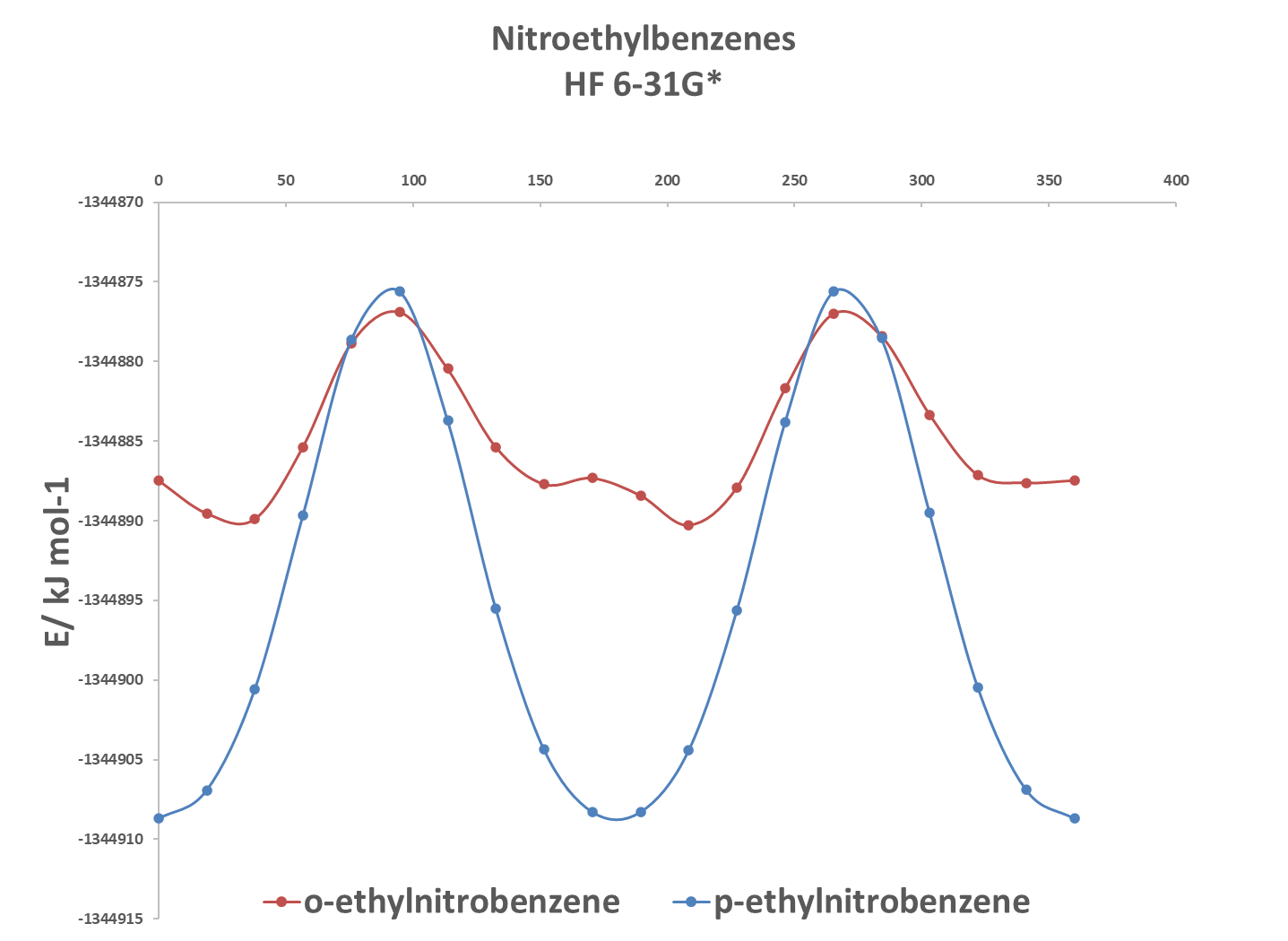
**

**
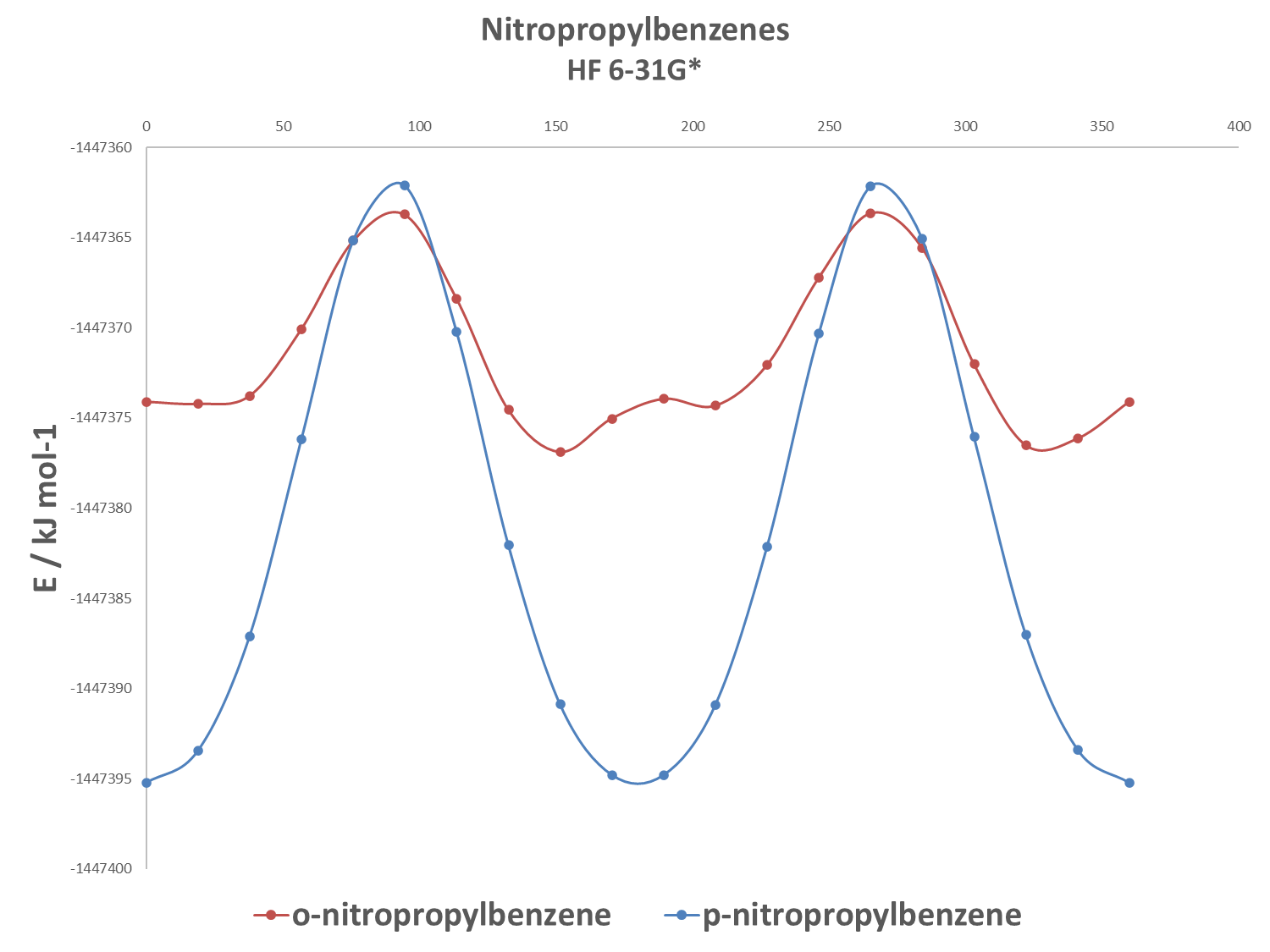
**

**
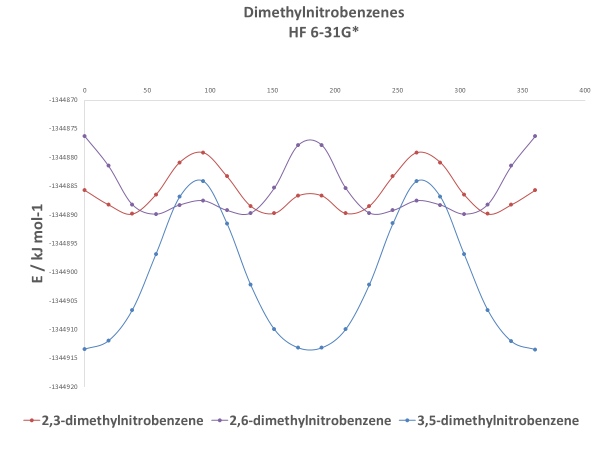
**

**Section 5: Deuterium NMR data**

*[methyl-D_3_]2,4-Dinitrotoluene*

This spectrum was recorded at 96%D, calculated for three atoms

*
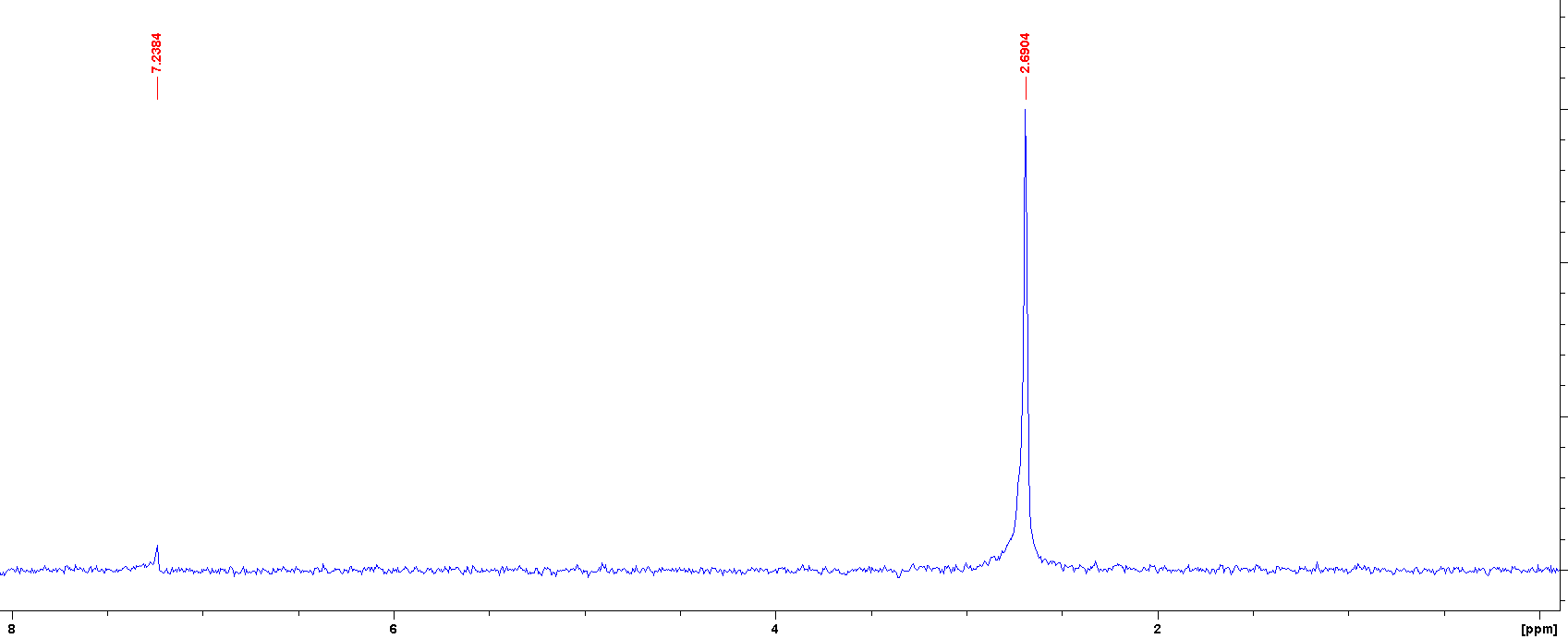
*

*[methyl-D]4-Nitrotoluene*

This spectrum was recorded at 79%D, calculated for three atoms


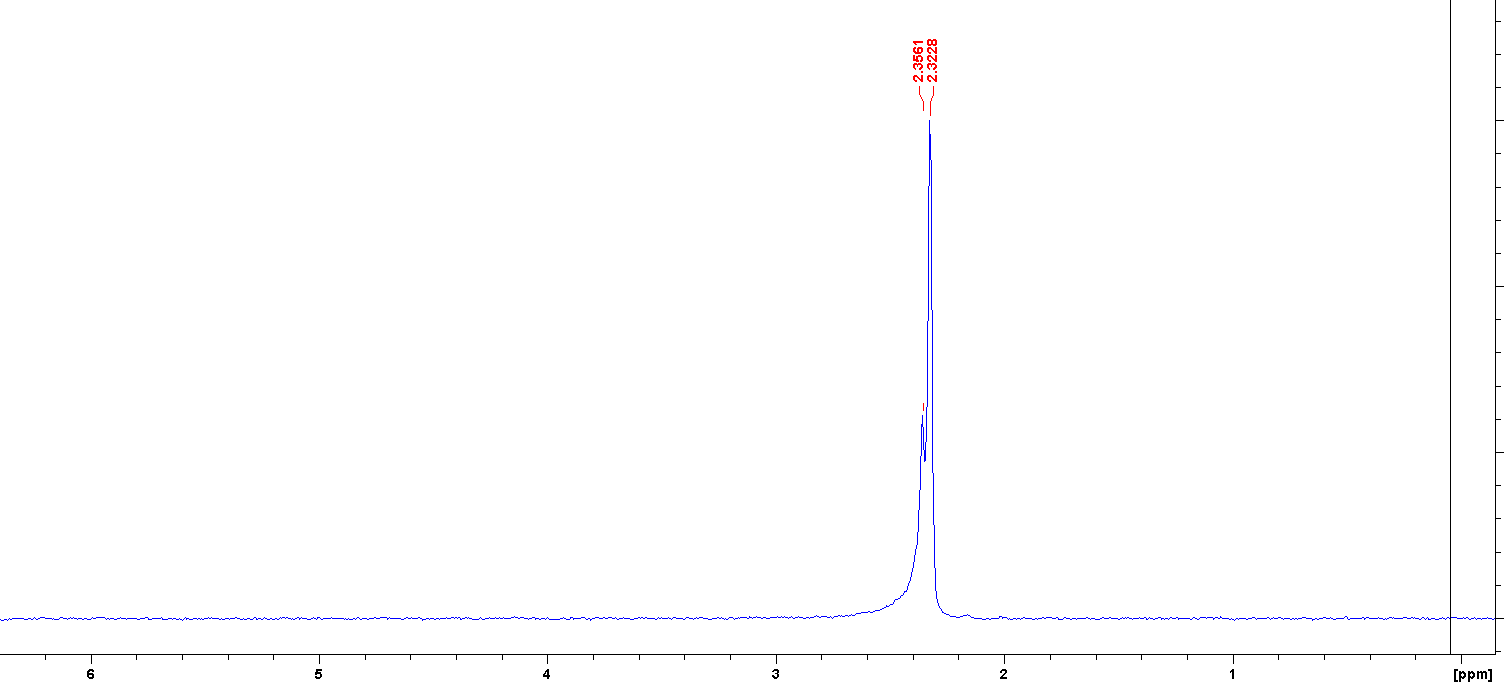


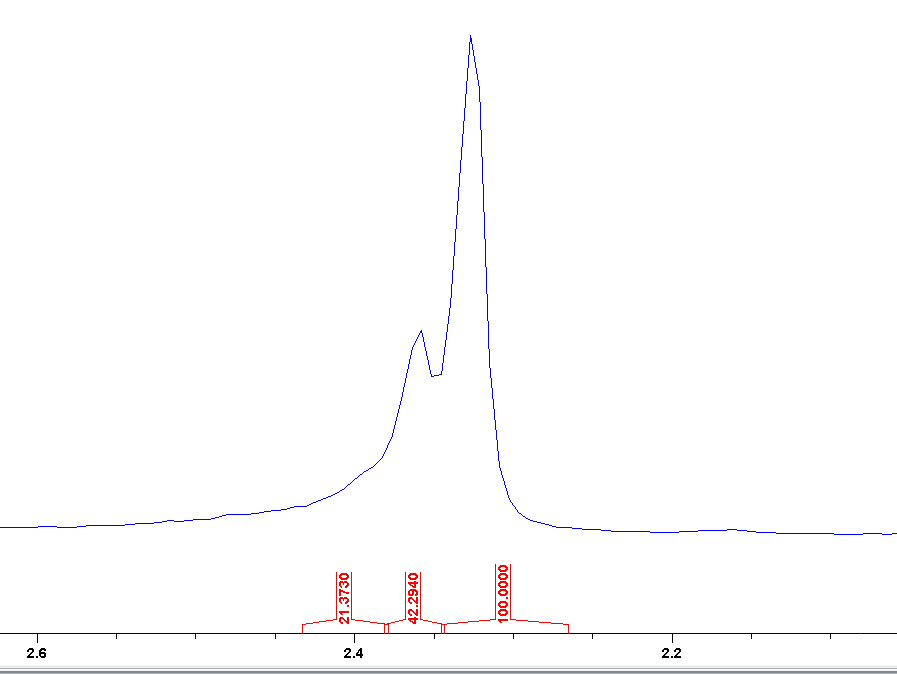


In this case there is a trace of CDH_2_, some CD_2_H and mainly CD_3_. Peaks are shifted towards high field by deuterium substitution. The α-isotope effect is ca. 0.33 ppm upfield for each deuterium substitution.

*[methyl-D]2-Nitrotoluene*

This spectrum was recorded at 16%D, calculated for three atoms and hence the main peak is CHD_2_ with lesser quantities of CH_2_D and CD_3_ at lower and higher chemical shifts.


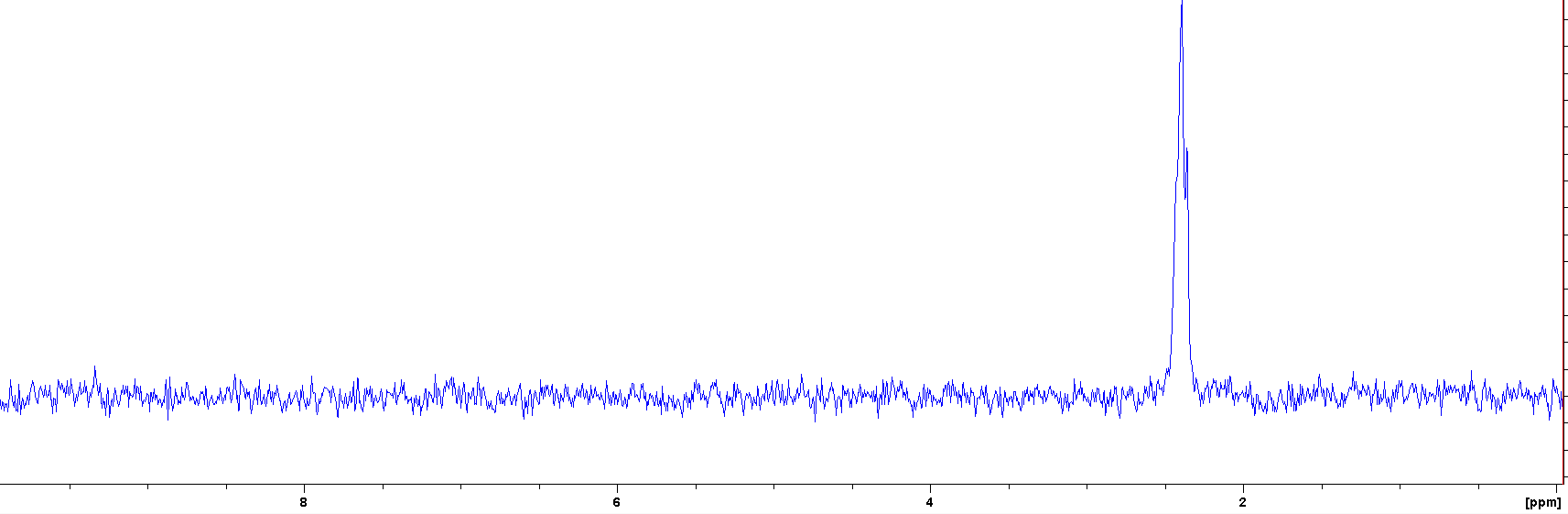


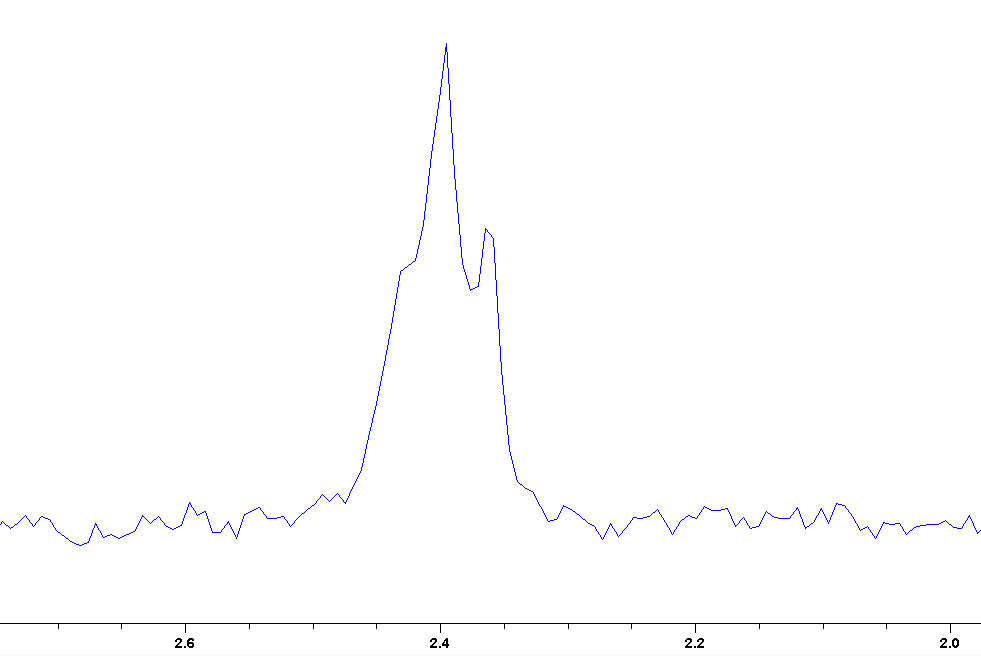


*4-[methylene-D]Ethylnitrobenzene*

This spectrum was recorded at 74%D, calculated for two atoms

*
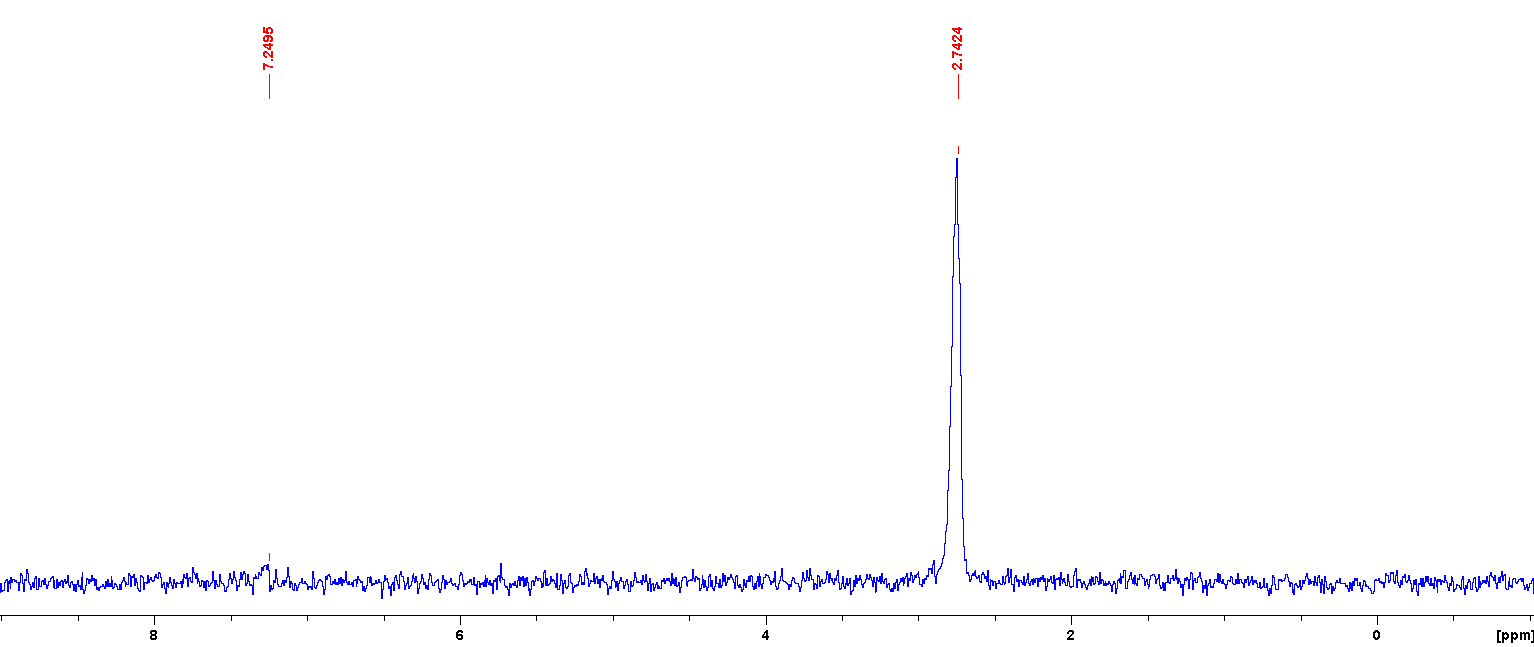
*

*
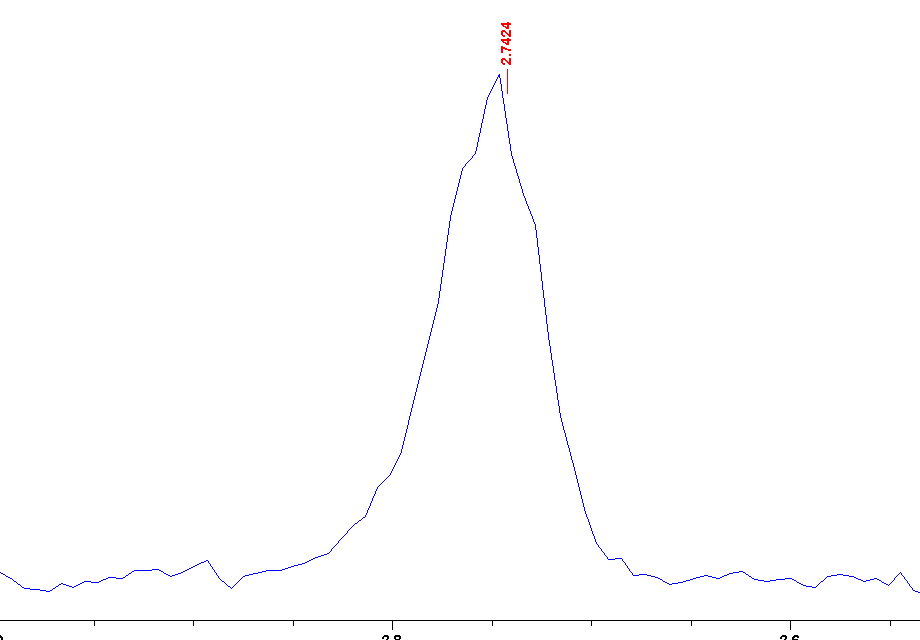
*

*2,4-[2,4-methyl-D]Dimethylnitrobenzene*

This spectrum was recorded at 52%D, calculated for 6 atoms

*
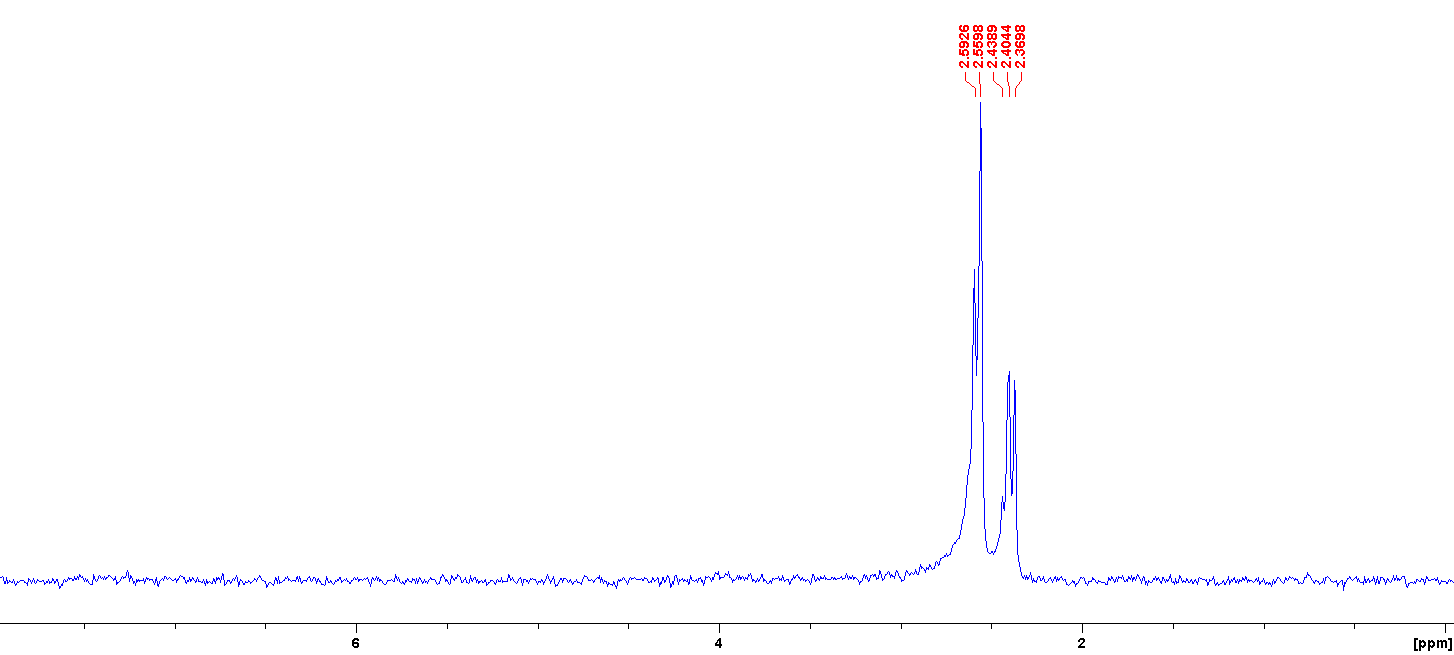
*

*
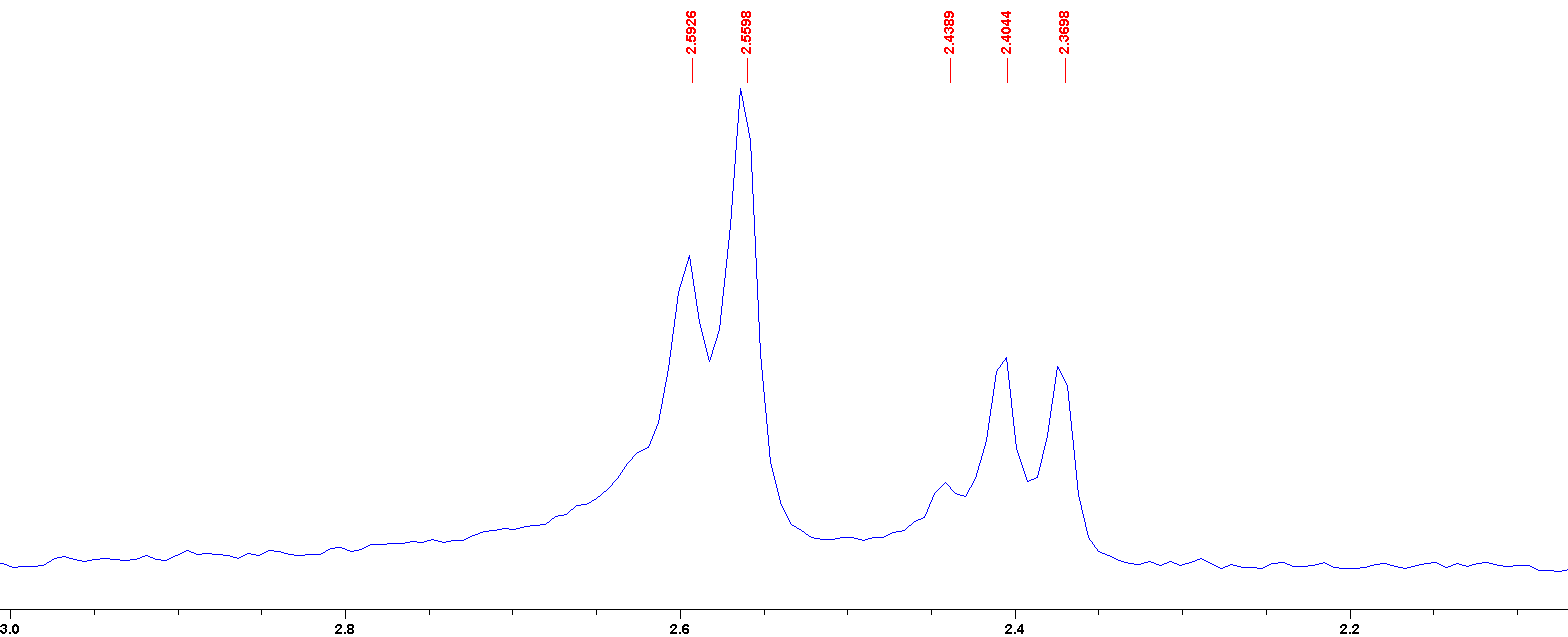
*

Here there are two labelled methyl groups. The most labelled methyl (*para* to NO_2_) is at ca. 2.6 ppm and is mainly CD_3_ with some CHD_2_, whilst the least labelled (*ortho*) at ca. 2.4ppm is mainly a mix of similar amounts of CHD_2_ and CD_3_ with some CH_2_D. Each deuterium substitution moves the methyl group towards TMS by *ca.* 0.34 ppm. CH_3_ species are not observed of course.

*
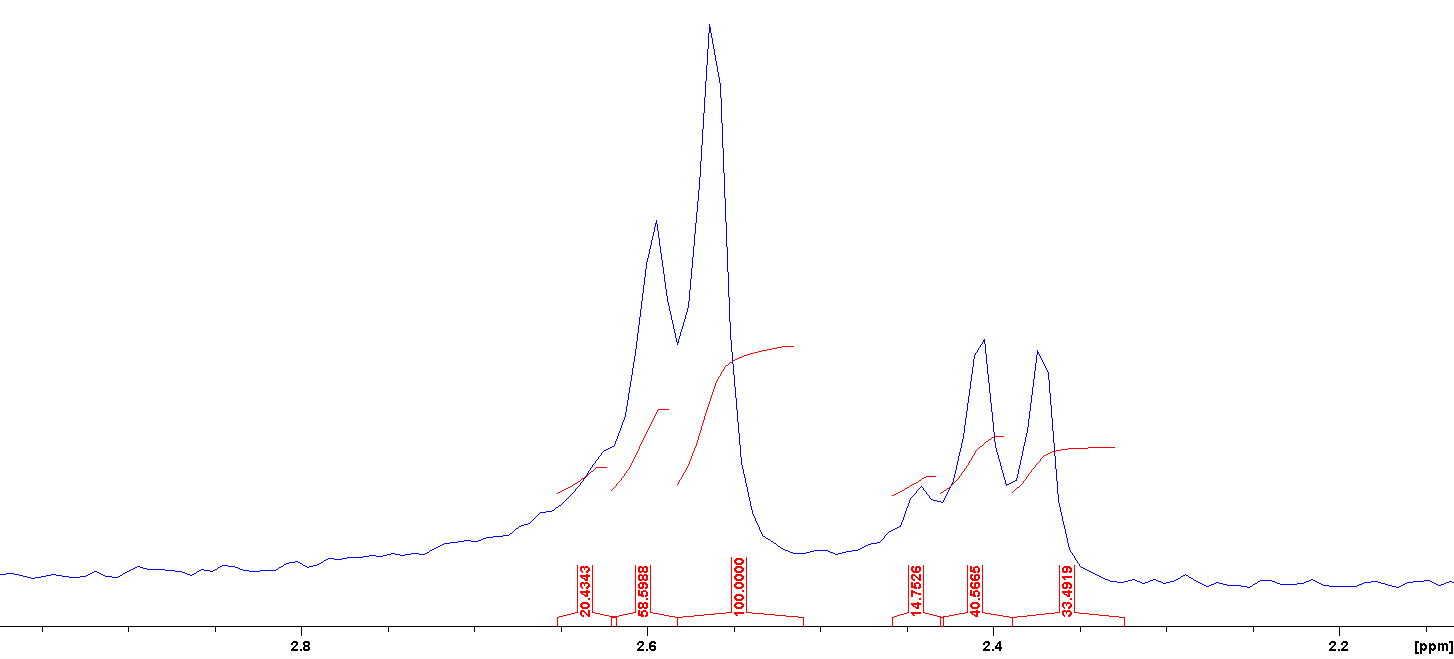
*

Section 6. Carbon-13 NMR data

*[methyl-D_3_]2,4-Dinitrotoluene,* Methyl region.

The small (2%) quantity of CHD_2_ (five lines) is over-represented because of the large Nuclear Overhauser Effect enhancement from its residual proton when compared with the major CD_3_ species (seven lines) which has no such enhancement.

*
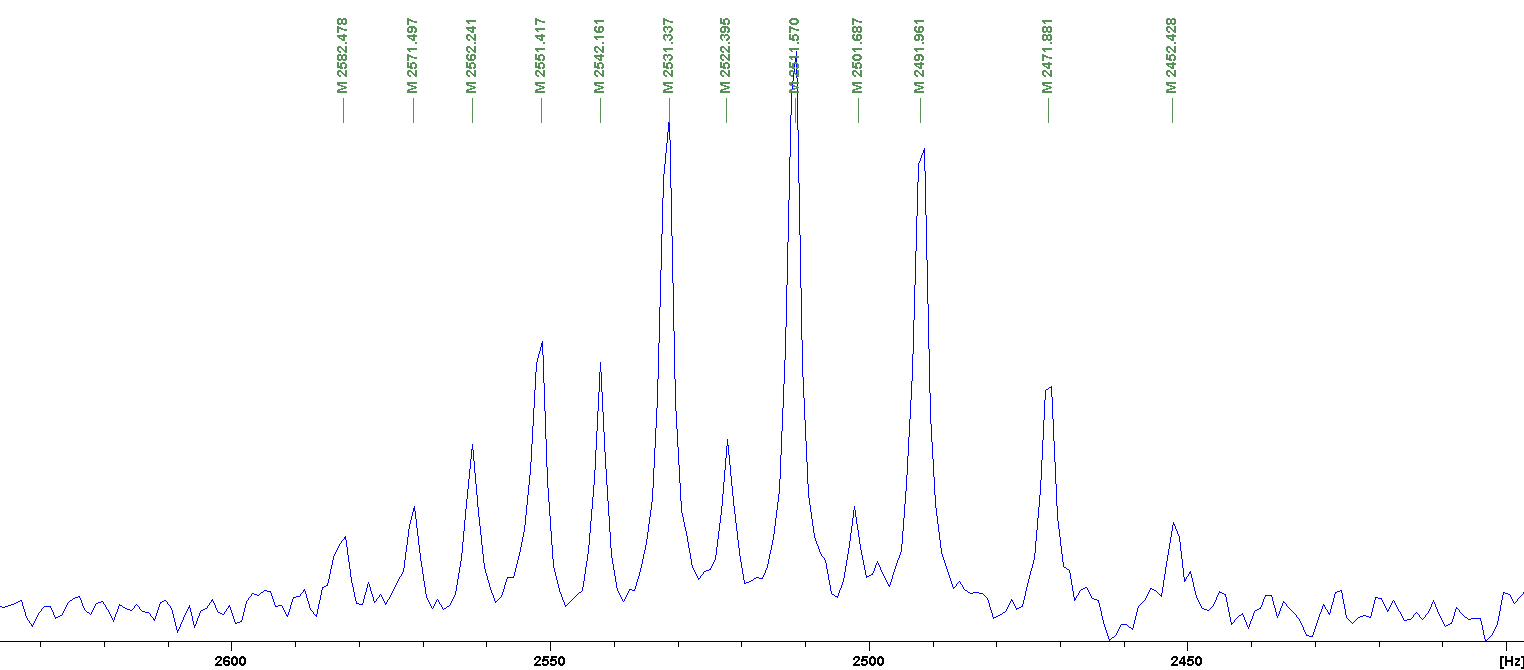
*

*[methyl-D]4-Nitrotoluene, Methyl region*

*
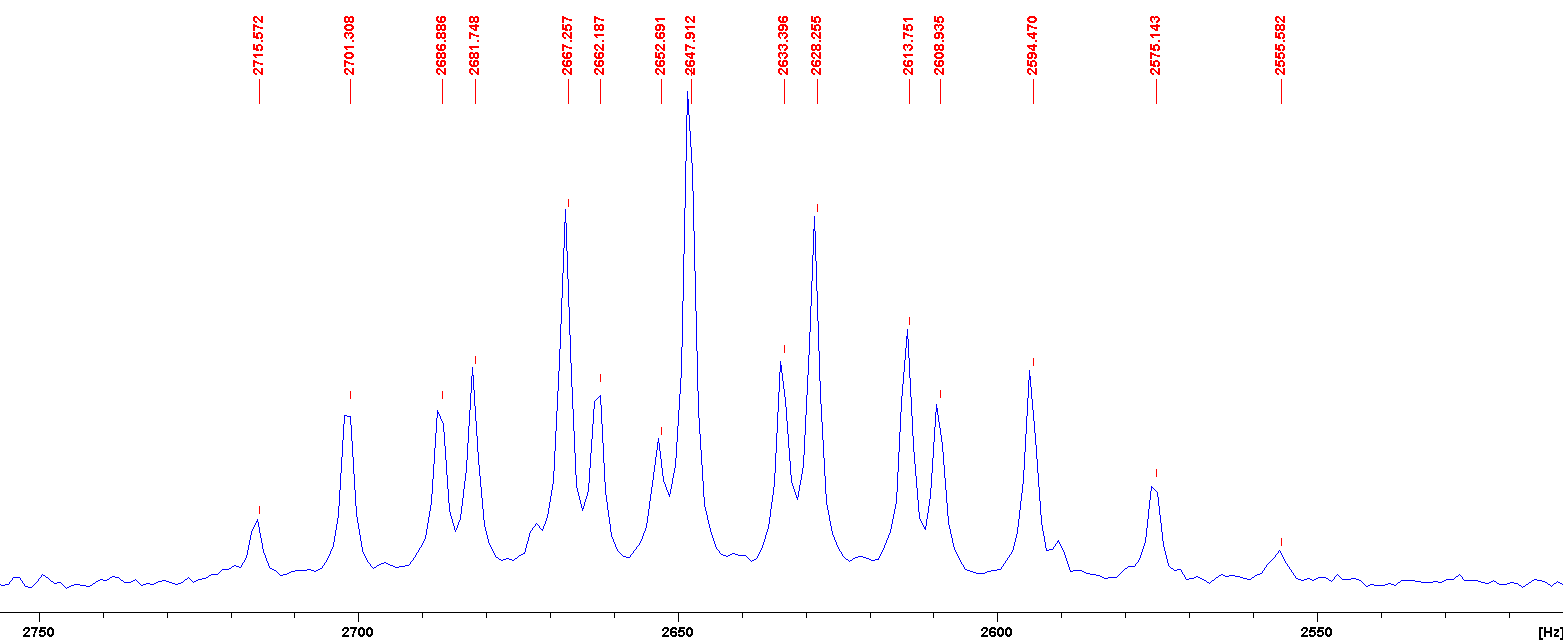
*

The species present form a triplet for CH_2_D, a pentuplet for CD_2_H and a heptuplet for CD_3_. Again those species bearing residual protons are over-represented because of large NOE enhancement. There is also a trace of a singlet CH_3_ at the furthest position downfield.

*[methyl-D]2-Nitrotoluene, Methyl region*

*
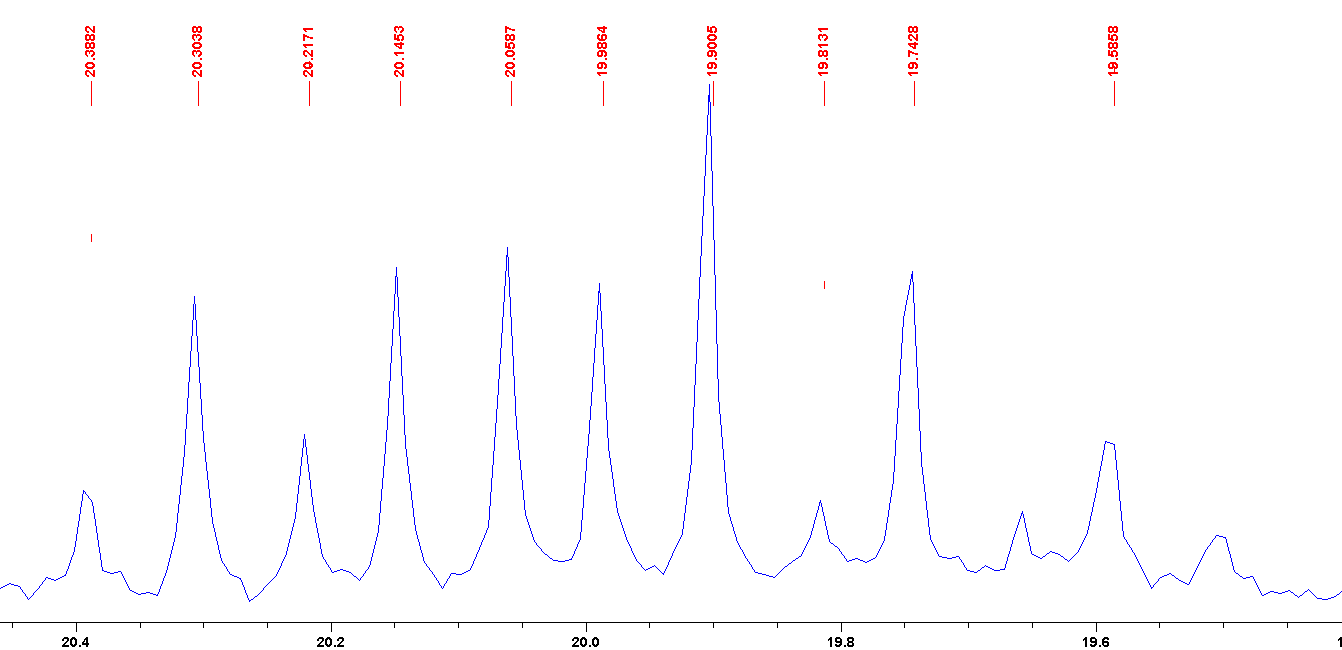
*

The analysis here is similar to that above.

*4-[methylene-D]Ethylnitrobenzene, methylene region CD2 + CHD*

*
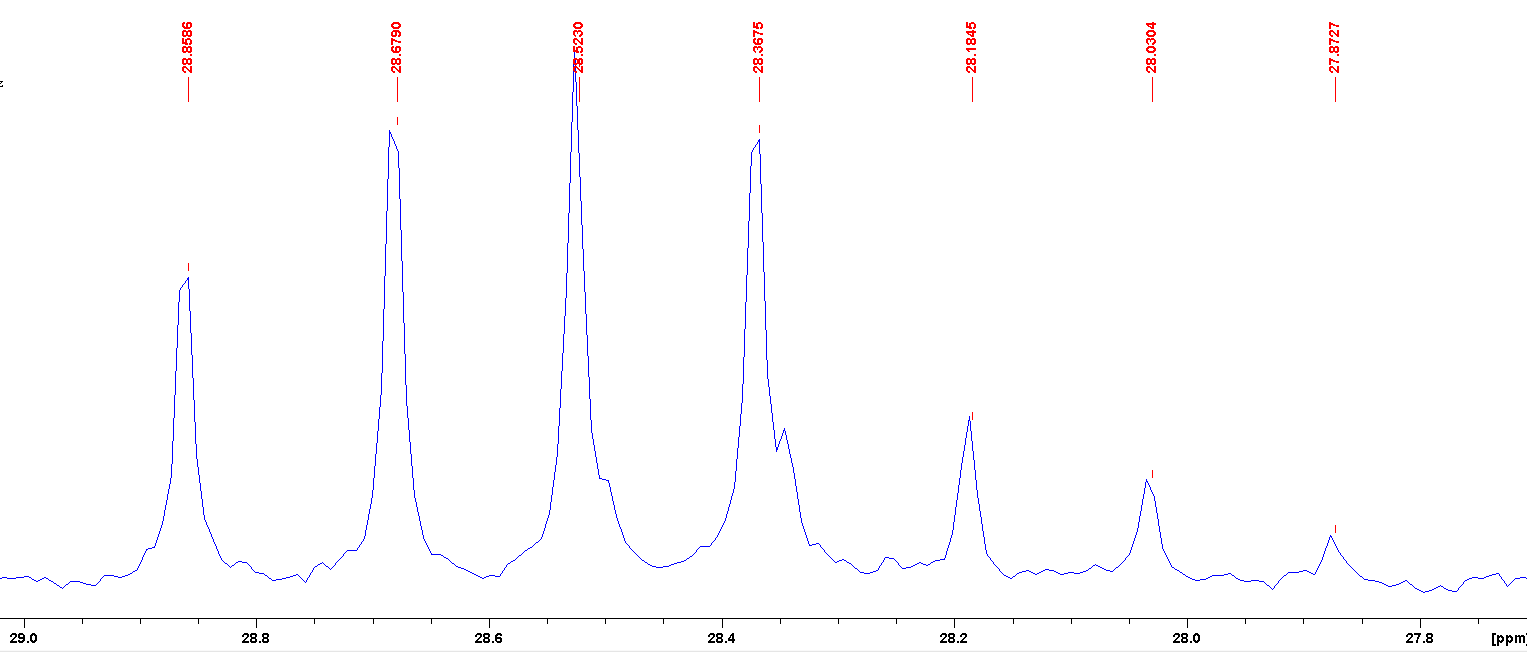
*

Here there is a singlet for CH_3_, a triplet for CDH_2_ *(ca.* 1:1:1), and a pentuplet for CD_2_ Again all species bearing protons are over-represented because of NOE enhancement. Each deuterium substitution moves the methyl resonance towards TMS by *ca*. 0.34 ppm.

*4-[methylene-D]Ethylnitrobenzene, Methyl region CH_3_CH_2_, CH_3_CHD and CH_3_CD_2_*

*
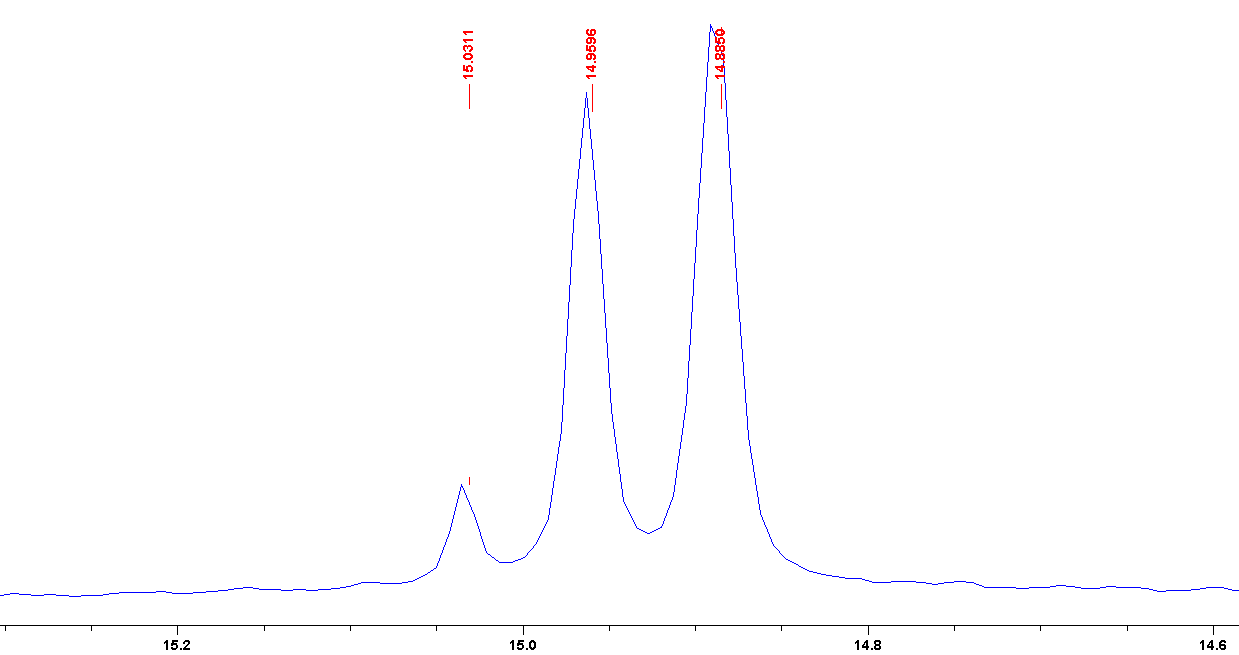
*

In this case the methyl groups are shifted upfield by a β-isotope effect arising from the adjacent labelled methylene group. Each deuterium substitution on the methylene moves the methyl resonance towards TMS by *ca.* 0.07ppm

*2,4-[2,4-methyl-D]Dimethylnitrobenzene, Methyls region*

*
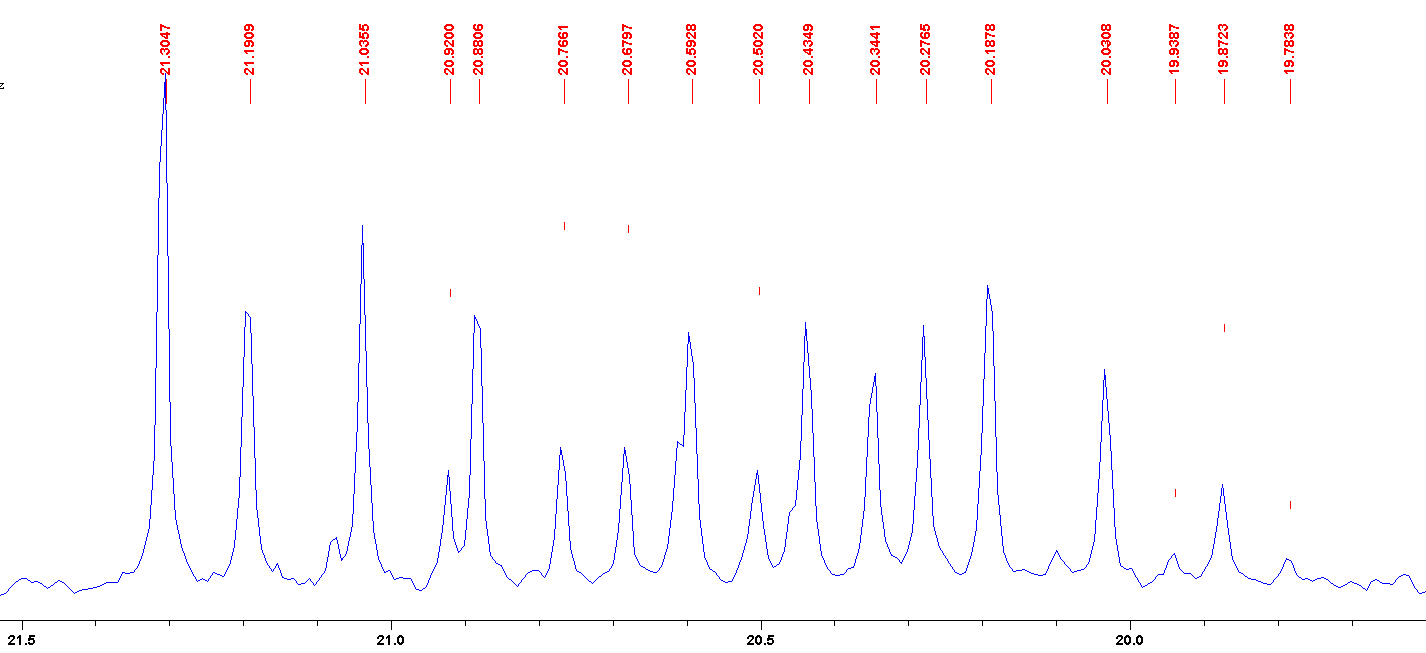
*

Again the intensities are strongly affected by the broad band decoupling of those species bearing protons, with the consequent NOE enhancement of such species compared with those containing more deuterium and fewer (or no) protons.

*Labelled methyl group 1:* 21.3047 CH_3_ singlet, 21.1909/21.0355/20.8806 CH_2_D,triplet, ca. 21,08/20.9200/20.7661/ca. 20.62/ca. 20.46 pentuplet CD_2_H

*Labelled methyl group 2:* 20.6797 CH_3_ singlet, 20.5928/20.4349/20.2765 triplet CH_2_D 20.5020/20.3441/20.1878/20.0308/19.8723 pentuplet CD_2_H, resonances at *ca.* 21.0, 19.5 and 17.9 could represent parts of the CD_3_ heptuplet
